# 17-DMAG regulates p21 expression to induce chondrogenesis *in vitro* and *in vivo*

**DOI:** 10.1242/dmm.033662

**Published:** 2018-10-08

**Authors:** Karri L. Bertram, Nadia Narendran, Pankaj Tailor, Christina Jablonski, Catherine Leonard, Edward Irvine, Ricarda Hess, Anand O. Masson, Saleem Abubacker, Kristina Rinker, Jeff Biernaskie, Robin M. Yates, Paul Salo, Aru Narendran, Roman J. Krawetz

**Affiliations:** 1 McCaig Institute for Bone and Joint Health, University of Calgary, Calgary, AB T2N 4N1, Canada; 2 Biomedical Engineering Graduate Program, University of Calgary, Calgary, AB T2N 4N1, Canada; 3 Department Cell Biology and Anatomy, University of Calgary, Calgary, AB T2N 4N1, Canada; 4 Department of Surgery, University of Calgary, Calgary, AB T2N 4N1, Canada; 5 Department of Chemical and Petroleum Engineering, University of Calgary, Calgary, AB T2N 4N1, Canada; 6 Centre for Bioengineering Research and Education, University of Calgary, Calgary, AB T2N 4N1, Canada; 7 Department of Comparative Biology and Experimental Medicine, University of Calgary, Calgary, AB T2N 4N1, Canada; 8 Alberta Children's Hospital Research Institute, University of Calgary, Calgary, AB T2N 4N1, Canada; 9 Division of Pediatric Oncology, Alberta Children's Hospital, Calgary, AB T3B 6A8, Canada

**Keywords:** Cartilage, p21, Mesenchymal stem cells, Regeneration, Macrophage

## Abstract

Cartilage degeneration after injury affects a significant percentage of the population, including those that will go on to develop osteoarthritis (OA). Like humans, most mammals, including mice, are incapable of regenerating injured cartilage. Interestingly, it has previously been shown that *p21* (*Cdkn1a*) knockout (p21^−/−^) mice demonstrate auricular (ear) cartilage regeneration. However, the loss of p21 expression is highly correlated with the development of numerous types of cancer and autoimmune diseases, limiting the therapeutic translation of these findings. Therefore, in this study, we employed a screening approach to identify an inhibitor (17-DMAG) that negatively regulates the expression of p21. We also validated that this compound can induce chondrogenesis *in vitro* (in adult mesenchymal stem cells) and *in vivo* (auricular cartilage injury model). Furthermore, our results suggest that 17-DMAG can induce the proliferation of terminally differentiated chondrocytes (*in vitro* and *in vivo*), while maintaining their chondrogenic phenotype. This study provides new insights into the regulation of chondrogenesis that might ultimately lead to new therapies for cartilage injury and/or OA.

## Abstract (precis)

**Summary:** p21 inhibition in response to 17-DMAG treatment induces chondrogenesis in human synovial mesenchymal stem cells and promotes auricular cartilage regeneration *in vivo*.

## INTRODUCTION

Approximately one in eight individuals suffer from osteoarthritis (OA), a disease characterized by degeneration of the cartilage surfaces of the joints with resulting pain and disability. Within a generation (30 years), there will be a new diagnosis of OA every 60 s (http://www.arthritisalliance.ca/images/PDF/eng/Initiatives/20111022_2200_impact_of_arthritis.pdf). As early as the 1700s, it was observed that the intrinsic regeneration capacity of articular cartilage is minimal (Hunter, 1995), and there is still a lack of approved therapeutic approaches proven to induce cartilage repair. Therefore, regenerative medicine approaches for cartilage repair offer a paradigm shift, which could fundamentally change health care delivery for patients suffering from cartilage injuries and/or OA.

Unlike most other tissues in the body, it is largely believed that articular cartilage does not contain a stem and/or progenitor cell population(s). Recent publications have challenged this dogma and suggested that such a population does exist in the superficial zone of articular cartilage and possesses the ability to undergo chondrogenesis (Jiang and Tuan, 2015; Yu et al., 2014). Nevertheless, it is clear that cartilage demonstrates an ineffective repair response after injury (Hunziker, 2002). It has long been assumed that the collagen matrix within the articular cartilage is static, with very little turnover occurring throughout adulthood, and a recent study by Heinemeier et al. supports this hypothesis (Heinemeier et al., 2016). Interestingly, however, in many mammals including humans, mesenchymal stem cell (MSC) populations exist both within the synovial membrane and synovial fluid (De Bari et al., 2001; Jones et al., 2004; Mochizuki et al., 2006; Yoshimura et al., 2007). In mouse and rabbit model systems, endogenous synovial MSCs can migrate to the site of cartilage injury and undergo chondrogenic differentiation *in vivo* (Hunziker and Rosenberg, 1996; Kurth et al., 2011). However, in these model systems, minimal repair of the cartilage is observed after injury. Recently, our group investigated the use of exogenous synovial MSCs to treat focal cartilage defects in mice, and observed that injection of these cells into an injured joint did confer some level of therapeutic benefit (Mak et al., 2016). Additionally, in that study, we also injected synovial MSCs derived from Murphy Roth's Large (MRL) mice [demonstrated to have an increased level of spontaneous injury repair (Clark et al., 1998; Diekman et al., 2013)], and found that MRL synovial MSCs display superior cartilage repair capacity compared with C57BL/6 synovial MSCs (Mak et al., 2016).

Mammals typically do not demonstrate cartilage repair after injury, although there are a few notable exceptions, such as the African Spiny mouse, which can almost completely regenerate ear cartilage injuries (Seifert et al., 2012). Although mouse pinna/auricular cartilage is elastic cartilage, it is similar to articular cartilage in the sense that ear cartilage does not spontaneously heal after injury (Clark et al., 1998). Interestingly, it has also been observed that MRL mice also have the capacity to regenerate articular cartilage after a focal defect (Fitzgerald et al., 2008). While the Spiny mouse and MRL mouse both demonstrate increased wound healing (including cartilage) after injury, these mice have a number of differences at the genetic and epigenetic levels compared with nonhealing strains (such as C57BL/6 mice) (Gawriluk et al., 2016). This makes it difficult to determine which gene(s) is responsible for the healer phenotype. Although a number of differentially expressed genes between healer and nonhealer strains have been identified, to our knowledge, only one of these genes has been shown to replicate the healing phenotype when knocked out. Specifically, Bedelbaeva et al. found that by knocking out *Cdkn1a* (*p21*), this resulted in a similar wound healing response (including increased chondrogenic regenerative ability) after ear punch injury (Bedelbaeva et al., 2010). Subsequent *in vitro* studies have demonstrated that p21 plays a role in stem cell differentiation, with knockdowns in bone marrow MSCs resulting in increased osteogenic and chondrogenic differentiation capacity (Yew et al., 2011). In an independent study using mouse induced pluripotent stem cells, it was demonstrated that knocking down p21 resulted in an enhancement of chondrogenic differentiation (Diekman et al., 2015). Furthermore, our own group has found a strong negative correlation between p21 expression levels and the ability of synovial MSCs to undergo effective chondrogenic differentiation (Masson et al., 2015). Taken together, this suggests that p21 plays a role in negatively regulating wound healing and chondrogenesis. Therefore, negatively regulating p21 expression could be a potential treatment option for enhancing chondrogenic differentiation in patients with cartilage injury and/or OA. However, p21 is a potent tumor suppressor (Georgakilas et al., 2017) and p21 knockout mice are not only at an increased risk of tumor development, but also demonstrate an increased risk of developing autoimmune disorders (Santiago-Raber et al., 2001; Topley et al., 1999). Therefore, the sustained inhibition of p21 would not be a realistic approach to increase wound healing and/or chondrogenesis, given the severe potential negative side effects. Thus, drug discovery approaches around p21 expression have focused on small molecules aimed at increasing the expression of p21 to inhibit tumor progression.

Therefore, in the current study, we undertook a drug screening and *in vitro* and *in vivo* validation approach to identify compounds that reversibly inhibit *p21* transcription/expression and assessed whether these compounds promote chondrogenic differentiation in human synovial MSCs. Once suitable compounds were identified and characterized *in vitro*, these were further examined in a mouse model of cartilage injury. Finally, we examined whether tissue resident MSCs and/or immune cells were playing a role within *in vivo* cartilage regeneration after drug treatment.

## RESULTS

### Identification of p21 expression inhibitors

#### Drug screening

**Fig. 1.**
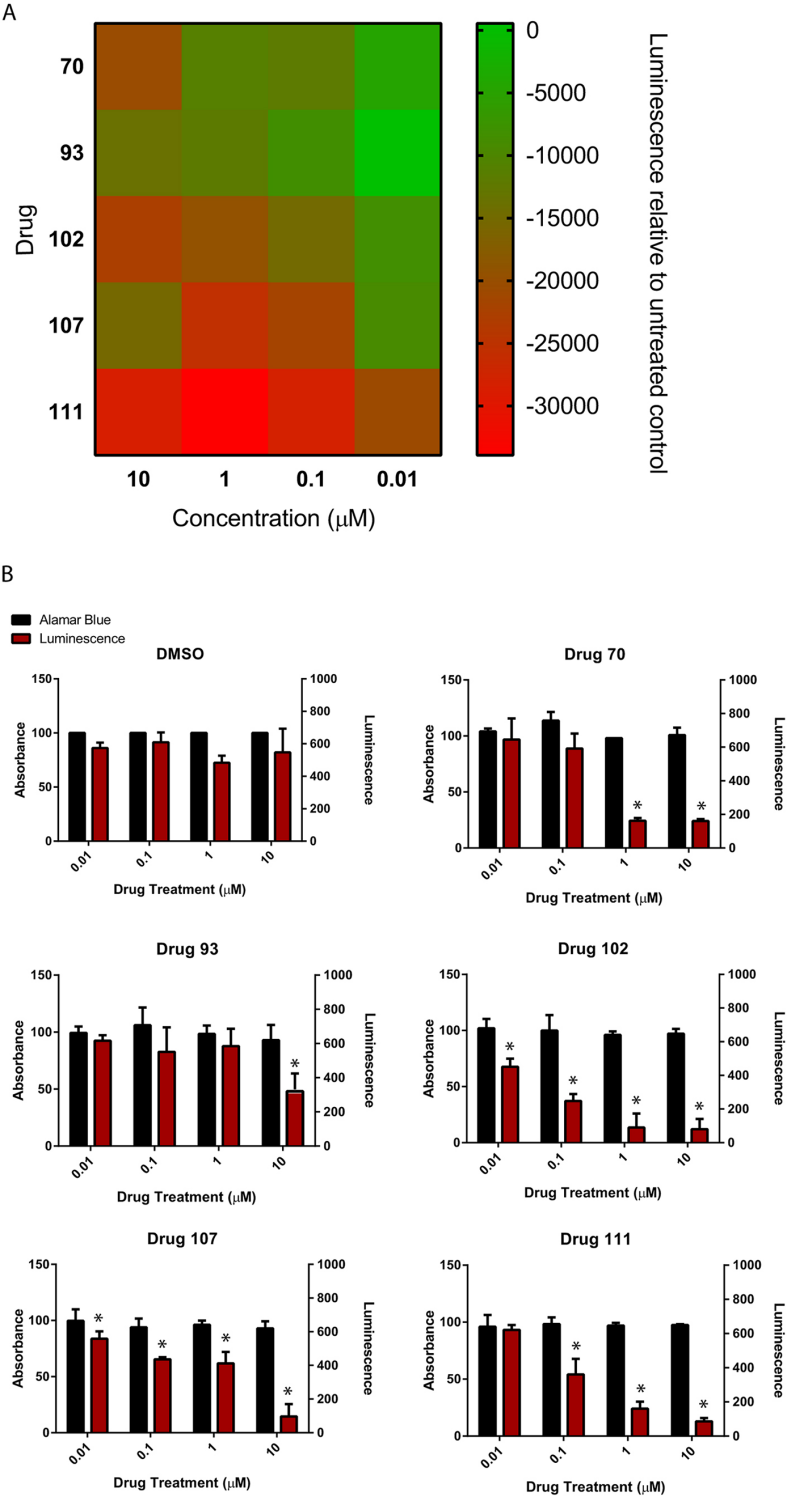
**Small molecule kinase inhibitors regulate p21 transcription in human cells.** (A) *p21* transcription decreased in p21 XMAN™ reporter cells after 24 h exposure to the five putative *p21* inhibitors in a concentration-dependent manner. (B) The five identified compounds did not induce metabolic toxicity in p21 XMAN™ reporter cells after 96 h treatment at the concentrations examined. At least four independent replicates were undertaken for each experiment. Data are mean±s.d.; *P<0.05.

**Fig. 2.**
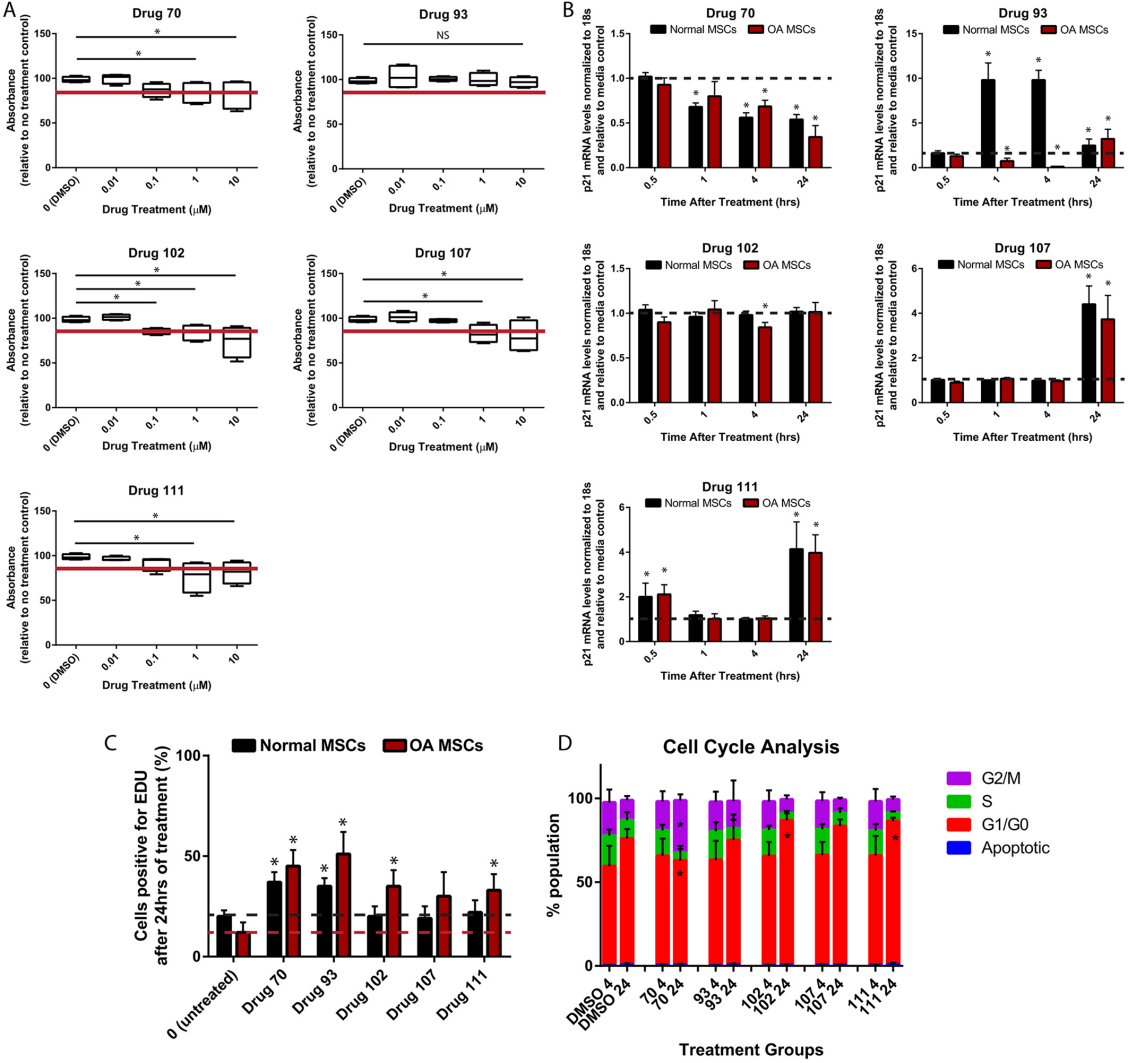
**Characterization of p21 regulators’ effects on human synovial MSCs.** (A) The five putative *p21* inhibitors did not cause metabolic toxicity in human MSCs after 24 h treatment at concentrations under 1 µM, with the exception of drug 93, which demonstrated no toxicity at any concentration examined. (B) *p21* mRNA levels were differentially affected by the different drugs in normal versus OA MSCs. Drug 70 decreased *p21* mRNA in both normal and OA MSCs, while drug 93 increased expression in normal and decreased expression in OA MSCs. Drug 102 also induced decreased *p21* mRNA expression only in OA MSCs, while drugs 102 and 111 increased expression in both normal and OA MSCs. (C) Only drugs 70 and 93 increased normal MSC proliferation, while drugs 70, 93, 102 and 111 were able to increase proliferation in OA MSCs. (D) Only drug 70 was able to increase the number of MSCs in the G2/M phase of the cell cycle, while an increase in G1/G0 accumulation was observed with drugs 102 and 111. At least three independent replicates were performed for each experiment (normal, *n*=5; OA, *n*=7). Data are mean±s.d.; NS, nonsignificant; *P<0.05.

Genetically modified HCT116 cells (XMAN™) expressing luciferase under the control of the p21 promoter were utilized in a high-throughput screen to identify potential p21-inhibiting compounds. A drug library of 146 small molecule compounds (Tables S1-S4) was selected for the initial screening. p21 XMAN™ reporter cells were exposed to each compound at four concentrations (0.01, 0.1, 1 and 10 µM) and the luminescence was measured after 24 h of treatment (Figs S1 and S2). From this initial screening, the five compounds that met the criteria of lowest luminescence, a concentration-dependent decrease in luminescence, and demonstrated no overt changes in cell morphology, cell death or cell detachment, were chosen for further testing (Fig. 1A). These will be referred to as drugs 70, 93, 102, 107 and 111. Their chemical names, their known pathways/mode of action (pathways inhibited) and their half maximal inhibitory concentration (IC50) according to the literature are summarized in Table S5. Additionally, four inhibitors that had previously been reported in the literature to inhibit p21 kinase activity [apocynin (Suzuki et al., 2013), SP600125 (Moon et al., 2011), olomoucine (King and Murphy, 2010) and butyrolactone I/IV (Sax et al., 2002)] were examined to determine whether they were able to decrease *p21* expression. None of these four drugs at any concentration tested proved to be effective at decreasing *p21* promoter activation (Fig. 2), nor were they able to induce chondrogenesis of synovial MSCs (data not shown); therefore, they were not included in further analyses.

#### Drug toxicity

To assess decreased luminescence in the initial screening was on account of increased cell death/decreased cell number; the metabolic toxicity of the five candidate *p21* expression inhibitors on the p21 XMAN™ reporter cells was assessed using an AlamarBlue assay (which measures metabolic activity of the cells) after 96 h of treatment. None of the five candidate *p21* expression inhibitors demonstrated a metabolically toxic effect on the p21 XMAN™ reporter cells over the range of concentrations assessed (Fig. 1B). Furthermore, luminescence was also re-validated in tandem with the metabolic toxicity analysis, and it was observed that all drugs induced a significant decrease in *p21*-driven luciferase expression that did not correspond with an increase in toxicity (Fig. 1B).

### Characterization of selected compounds on synovial MSCs

#### Drug toxicity

The five putative *p21* expression inhibitors were then examined in the relevant cell of interest, human synovial MSCs. The metabolic toxicity was assessed using AlamarBlue to detect the metabolic activity of the cells after drug treatment (Fig. 2A; Table S6). As there is no universal acceptable limit for cell toxicity, the cutoff used for this experiment was 85% of the carrier control. Drugs 70, 102, 107 and 111 dropped below this viability cutoff at concentrations higher than 0.1 µM in the human synovial MSCs. Drug 93 did not drop below this cutoff. Therefore, for the remainder of the experiments in human synovial MSCs, cells were treated with 0.1 µM drugs 70, 102, 107 and 111, and 10 µM drug 93.

#### *p21* gene expression

To determine whether these drugs could reduce *p21* expression levels in human synovial MSCs in addition to the XMAN™ reporter cells, *p21* mRNA was assessed in human synovial MSCs derived from both normal individuals and OA patients at four different time points of drug treatment over 24 h (0.5, 1, 4 and 24 h) (Fig. 2B). Normal and OA synovial MSCs were separated, because we have previously demonstrated that there is a significant difference in basal p21 expression (mRNA and protein), with normal MSCs demonstrating significantly lower expression levels of p21 in an undifferentiated state (Masson et al., 2015). When the *p21* mRNA levels were assayed after drug treatment, it was observed that, in most cases, normal and OA MSCs responded in a similar fashion. Specifically, drug 70 reduced *p21* mRNA expression within 1 h and this was still observed at 24 h post-treatment. Drug 102 decreased *p21* expression at 4 h, but only in OA MSCs. Drug 107 did not decrease *p21* mRNA levels, but instead increased *p21* expression at 24 h in both normal and OA MSCs. Drug 111 also increased *p21* expression at 30 min and 24 h after treatment in normal and OA MSCs, but, interestingly, no effect was observed at 1 h or 4 h after treatment. The effects of drug 93 on synovial MSCs were quite interesting, as it decreased *p21* expression in OA MSCs only at 1 h and 4 h after treatment, but increased *p21* expression in normal MSCs at the same time points. At the protein level, similar (but not identical results) were observed. Dimethyl sulfoxide (DMSO) treatment had no effect on p21 protein levels, whereas drug 70 and drug 111 decreased p21 levels in OA, but not normal synovial MSCs. Drugs 93 and 102 had no effect on p21 protein levels in OA or normal MSCs at the time points examined, whereas drug 107 increased p21 levels in normal MSCs (Fig. S3).

#### Cell proliferation

Because p21 is a cell cycle inhibitor, acting mainly at the G1/S checkpoint to impede cells from dividing until they have undergone proofreading (Georgakilas et al., 2017), the proliferation and cell cycle was analyzed in synovial MSCs while exposed to these potentially p21-inhibiting compounds. Again, normal and OA synovial MSCs were analyzed separately, because we previously demonstrated differences in proliferation between normal versus OA. Proliferation of the human synovial MSCs was analyzed after 24 h of drug treatment using an EdU assay (Fig. 2C). Drugs 93 and 70 demonstrated a significant increase in proliferation compared with the DMSO control in normal and OA MSCs, whereas drugs 102, 107, and 111 did not show any change in proliferation after 24 h of treatment in normal MSCs, but drugs 102 and 111 increased the proliferation of OA MSCs (Fig. 2C).

#### Cell cycle regulation

The distribution of human synovial MSCs within the cell cycle with drug treatment was analyzed using a propidium iodide assay. Human synovial MSCs were treated for 4 h and 24 h with each drug and then analyzed using flow cytometry (Fig. 2D). The data are summarized in Fig. S4, including serum starvation and nocodazole (Apraiz et al., 2017) treatments as controls for each drug. All treatment groups except drug 70 showed an increase in G1/G0 accumulation after the 24 h treatment, compared with the DMSO control at 4 h. Drug 70 showed an increase in G2/M accumulation compared with the DMSO control after 24 h of treatment, whereas drugs 102 and 104 significantly increased G1/G0 accumulation compared with the same control (Fig. 2D). Because inhibition of p21 allows cells to freely enter the cell cycle, a p21 expression inhibitor should allow the accumulation of cells in G2/M, and this has been observed as a phenotype in fibroblasts from p21^−/−^ mice (Bedelbaeva et al., 2010).

### Effect of p21-inhibiting drugs on human synovial MSC chondrogenesis

**Fig. 3.**
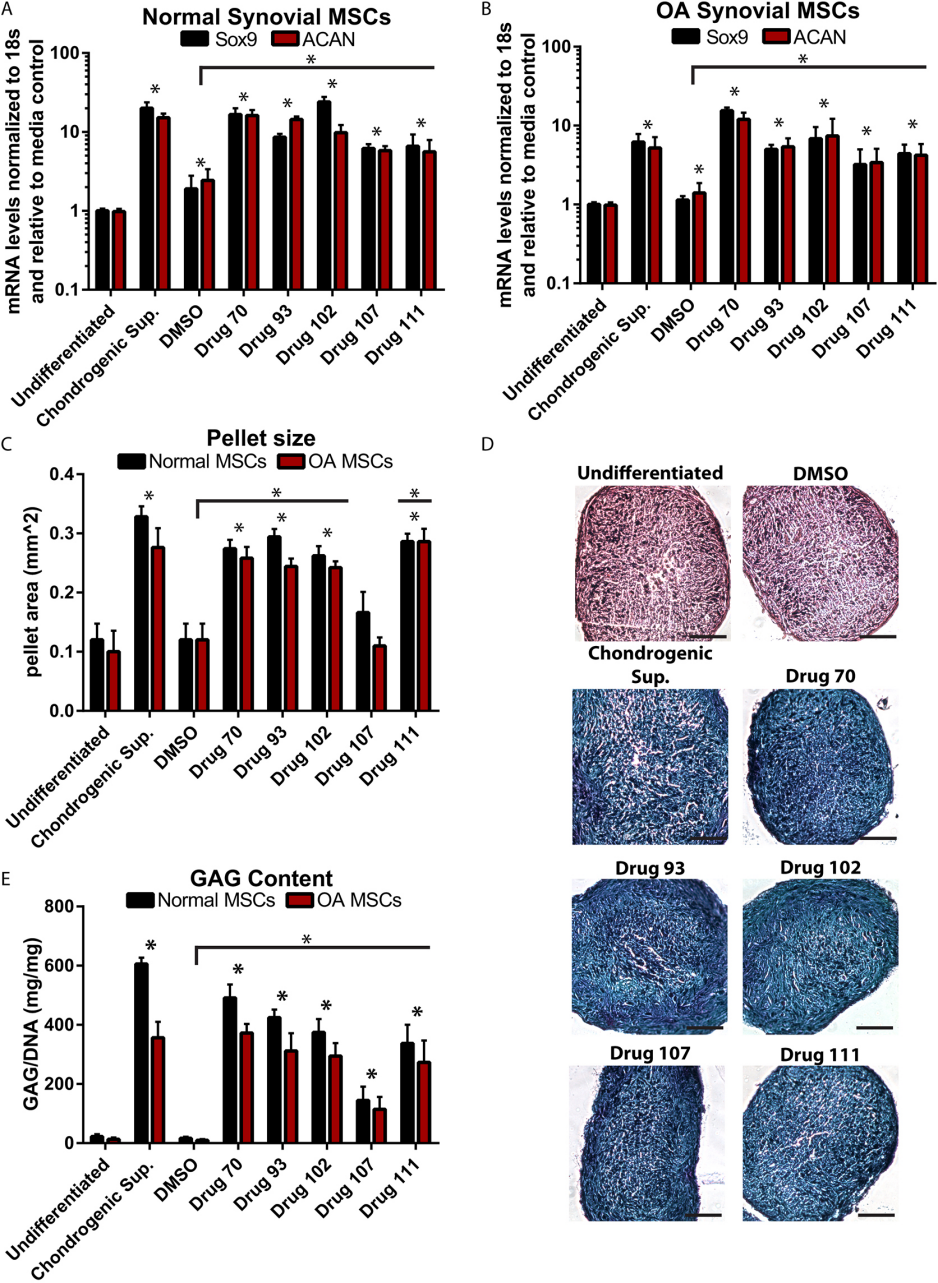
**Characterization of p21 inhibitors’ effects on human MSC chondrogenesis.** (A,B) For all drugs examined (and positive controls), the chondrogenic markers *SOX9* and *ACAN* were increased in both normal and OA MSCs after treatment. (C) Furthermore, all drugs (except for 107) increased the pellet size following chondrogenic differentiation. (D,E) Additionally, all drug treatments resulted in pellets that demonstrated Alcian Blue staining (D) and demonstrated increased GAG content over controls (E). At least three independent replicates were performed for each experiment (normal, *n*=5; OA, *n*=7). Data are mean±s.d.; *P<0.05. Scale bars: 50 μm.

The putative p21 expression inhibitors were then analyzed to determine whether they were able to induce chondrogenesis in human synovial MSCs derived from normal individuals and OA patients. Human synovial MSCs were pelleted and cultured in standard (without chondrogenic factors) medium containing the drug treatment for 21 days. Chondrogenic marker expression was assessed using reverse transcription quantitative PCR (RT-qPCR). *SOX9* and *ACAN* were upregulated by chondrogenic supplements, and drugs 70, 93, 102, 107 and 11 in both normal (Fig. 3A) and OA (Fig. 3B) synovial MSCs. Interestingly, DMSO also increased the expression of *SOX9* and *ACAN*, but not to the level of the chondrogenic supplements, nor the drug treatments. Pellet size was assessed because it is an indicator of both cell growth and extracellular matrix (ECM) deposition, which are known to occur at the beginning stages of chondrogenic differentiation (Yamashita et al., 2009). Compared with the undifferentiated control (cells pelleted, but no chondrogenic factors, nor drug treatment), cells pelleted with chondrogenic growth factors (chondro, positive control), and drugs 70, 93, 102 and 111 demonstrated a significant increase in pellet area (Fig. 3C), whereas no increase was observed with drug 107 or DMSO treatment. Because an increase in pellet size can be nonspecific and solely indicate an increase in cell number or more ECM deposition, Alcian Blue was used to stain the pellets for proteoglycan content. The undifferentiated and DMSO treatment pellets were negative for Alcian Blue staining, whereas cells treated with chondrogenic supplements, or drugs 70, 93, 102, 107 or 111, all demonstrated positive staining for Alcian Blue (Fig. 3D). Lastly, to confirm the presence of glycosaminoglycans (GAGs) suggested by Alcian Blue staining, total GAG content was quantified. Quantification showed that all drug treatments and the chondrogenic supplements increased GAG content within the pellets, whereas DMSO treatment did not induce GAG production (Fig. 3E). The results from all chondrogenic/pellet size assays were also re-evaluated and compared with the DMSO carrier control, to confirm that the respective drug, and not DMSO, was responsible for the effect observed.

Although all of the drugs tested were able to induce the chondrogenic differentiation in human synovial MSCs and are worthy of further study, only drug 70 was chosen to move forward with the *in vivo* experiments because exposure of MSCs to this drug led to (1) an increase in proliferation, (2) an accumulation of the MSCs in the G2/M phase and (3) induction of chondrogenic differentiation within normal and OA human synovial MSCs.

### *In vivo* cartilage generation

**Fig. 4.**
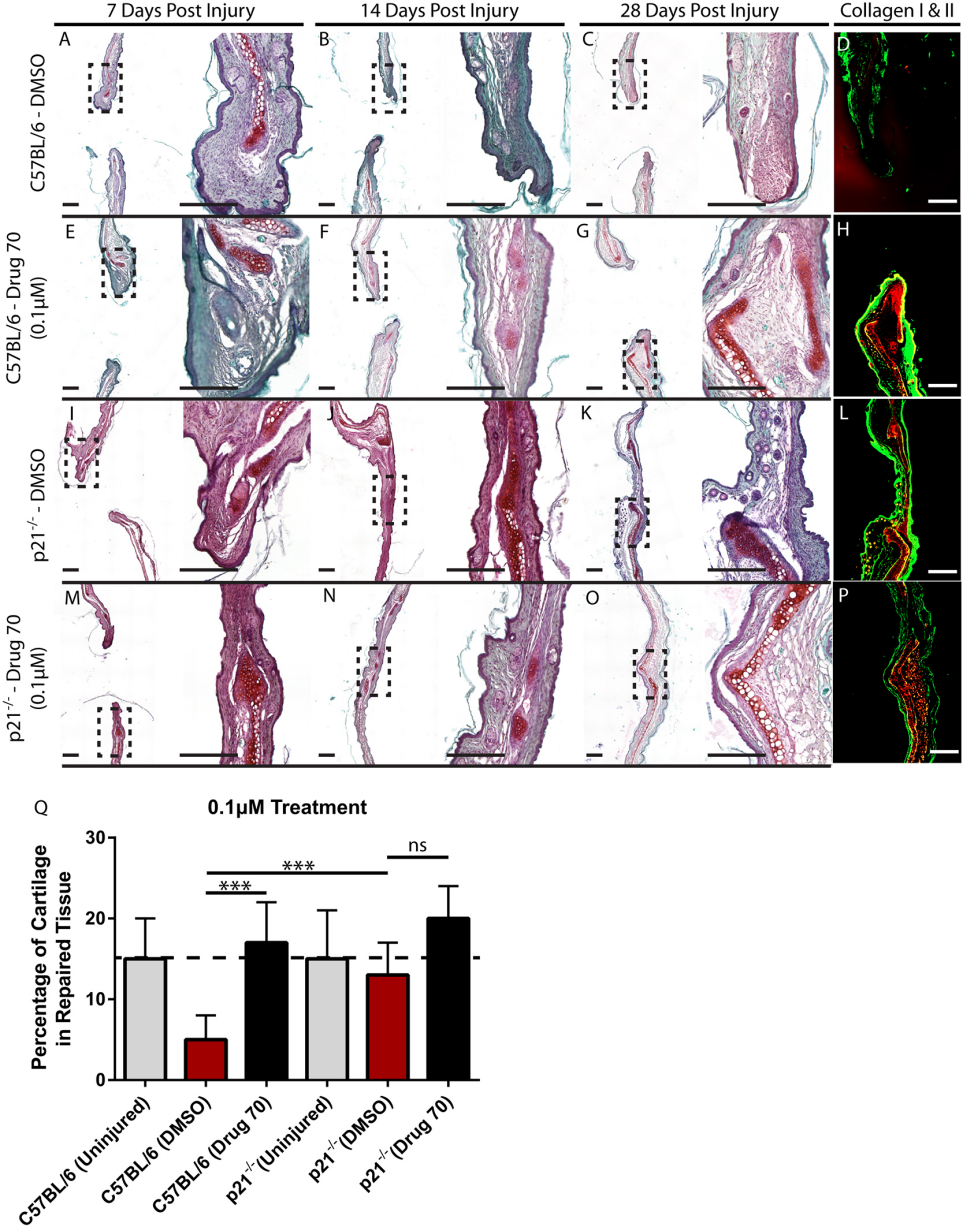
**Characterization of drug 70** ***in vivo* in a mouse model of cartilage injury.** (A-C) C57BL/6 and p21^−/−^ mice were treated with DMSO or drug 70 and examined at 7, 14 and 28 days postinjury with Safranin O staining. In DMSO-treated C57BL/6 mice, little cartilage (bright red tissue) within the injury site was observed. (E-G) However, with drug 70 treatment (0.1 µM), cartilage could be observed within the defect site at all time points examined. (I-K) In p21^−/−^ mice treated with DMSO, at 28 days postinjury, the wound was almost completely closed and a new cartilage scaffold was present. (M-O) Following drug 70 treatment, the injury site demonstrated near complete regeneration and integration with the native tissue. (D,H,L,P) At 28 days postinjury, the Safranin O staining was validated with collagen type I (green) and collagen type II (red) staining. (Q) The amount of cartilage tissue was quantified within the treatment groups and it was found that drug 70 significantly increased cartilage formation after injury to similar levels as those observed in DMSO-treated p21^−/−^ mice. Scale bars: 200 µm. Data are mean±s.d.; ns, nonsignificant; ***P<0.01.

As it has been previously demonstrated that there is an extremely high correlation in the ability to heal articular and auricular cartilage injuries in mice (Rai et al., 2012), we chose to examine the effects of the HSP90 (HSP84-2) inhibitor (drug 70) *in vivo*, a mouse auricular cartilage injury model, because this would facilitate drug delivery and allow for a repeated measures approach. Through-and-through (2 mm) ear punches were performed on control nonhealing C57BL/6 mice. The wound was locally treated with 0.1 µM drug 70 (*n*=15/5 per time point) once a week for 4 weeks. DMSO-treated control (*n*=15/5 per time point) mice were used as a negative control, and DMSO-treated p21^−/−^ mice (*n*=30, *n*=15 per treatment group/five per time point) were used as a positive control for wound healing. The ear tissue was harvested after sacrificing the mice at 7, 14 and 28 days after injury and stained with Safranin O/Fast-Green and collagen type I and II to visualize the layer of cartilage tissue within the ear. The C57BL/6 DMSO-treated control sections showed little evidence of cartilage repair after injury over the time points examined (Fig. 4A-D), whereas in the C57BL/6 drug 70-treated animals, extensive cartilage formation was observed within the wound area. Specifically, new cartilage can be observed developing adjacent to the original damaged cartilage (Fig. 4E-H). In p21^−/−^ mice, the wound area is almost completely healed by 28 days postinjury and new cartilage tissue can be seen within the injury site, which has almost bridged the gap across the original injury site (Fig. 4I-L). In drug 70-treated p21^−/−^ mice, we observe a synergistic effect, where the cartilage has completely bridged the original injury site and closely resembles the native tissue by 28 days postinjury (Fig. 4M-P). To quantify the amount of cartilage tissue produced in the wound site, the surface area of cartilage within the wound site was normalized to the area of the wound site at 28 days postinjury (Fig. 4Q). In a normal uninjured mouse ear (C57BL/6 or p21^−/−^), the cartilage represents ∼15% of the ear cross-sectional surface area. By 28 days postinjury in C57BL/6 mice treated with DMSO, cartilage represents only ∼5% of the wound site. In C57BL/6 mice treated with drug 70, there is an increase in cartilage compared with DMSO controls. There was no difference in cartilage content between uninjured p21^−/−^ mice or p21^−/−^ mice treated with DMSO or drug 70 (Fig. 4Q).

To determine whether a dose response relationship exists between drug treatment and *in vivo* cartilage formation exists, the experiment was repeated with a higher concentration of drug 70 (100 µM) in C57BL/6 (*n*=8) and p21^−/−^ (*n*=8) mice (Fig. S5A). No difference was observed between 0.1 µM versus 100 µM drug 70 treatment in terms of new cartilage surface area. However, in both C57BL/6 (Fig. S5B) and p21^−/−^ (Fig. S5C) mice treated with 100 µM, robust cartilage formation was observed, although abnormal tissue morphology was also observed, and in the case of p21^−/−^ mice, wound healing was negatively impacted. Therefore, for the following experiments, only the 0.1 µM drug concentration was examined.

### Effect of drug 70 treatment on tissue-resident MSCs

#### C57BL/6 mice

**Fig. 5.**
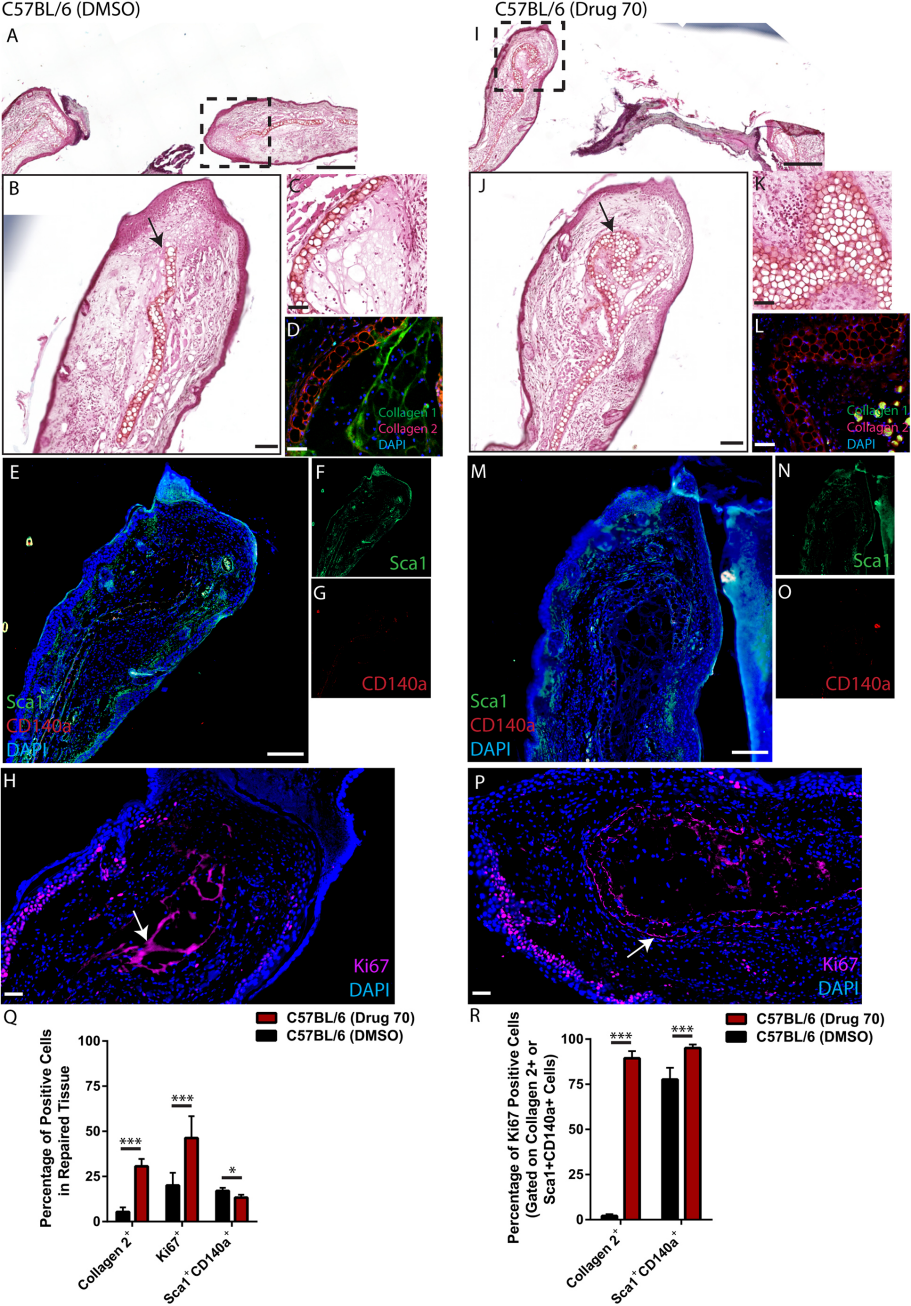
**Characterization of cells** **expressing MSC markers 3 days postinjury.** (A,B) Injured C57BL/6 mice treated with DMSO demonstrated little to no cartilage at the site of injury (arrow). (C,D) Fibrotic-like tissue was observed adjacent to the cartilage scaffold (C) that stained positive for collagen type I (D). (E-G) Cells expressing Sca1 (F), CD140a (G) or both (E) were present within the wound site. (I,J) With drug 70 treatment, a large amount of new cartilage could be observed within the wound site (arrow). (K,L) Fibrotic-like tissue was not observed adjacent to the cartilage scaffold (K) with absence of collagen type I staining (L). (N-P) Sca1 (N), CD140a (O) or double-positive cells (P) were present. In DMSO-treated animals, Ki67-positive cells were observed within the epidermis and also within an area of fibrotic tissue (arrow, H), while in drug 70-treated animals, Ki67 staining was present within the epidermis and cartilage scaffold of the ear (arrow, P). (Q,R) Flow analysis of the wound area in both DMSO- and drug 70-treated animals demonstrated that significantly more chondrocytes (collagen type II^+^) were present with drug treatment (Q), and that almost all chondrocytes were proliferating *in vivo*, compared with almost no proliferating chondrocytes observed with DMSO treatment (R). Data are mean±s.d; *P<0.05, ***P<0.01. Scale bars: 200 µm in A and I; 50 µm in B-D, H, J-L and P; 100 µm in E and M.

To investigate the cellular mechanism behind the increased cartilage observed in drug 70-treated animals, earlier time points were examined. C57BL/6 mice (*n*=5) were injured and treated with 0.1 µM drug 70, then sacrificed 3 days postinjury. Interestingly, in the drug 70-treated animals (Fig. 5B), a large amount of tissue/wound infiltrate was observed, and this was not found in DMSO-treated groups (Fig. 5A); however, this tissue appeared to be transient/unstable as it was not seen at day 7 postinjury or later time points. At 3 days postinjury, the cartilage scaffold of the injured ear was observed in DMSO-treated animals and abruptly ended just before the site of the initial wounding (Fig. 5A,B). Fibrotic-like tissue was also observed adjacent to the cartilage scaffold (Fig. 5C), and this was confirmed with collagen type I and II staining (Fig. 5D). Interestingly, with drug 70 treatment, a significantly larger cartilage layer can be observed that is folded over on itself and represents most of the tissue present at the injury site (Fig. 5I,J). Little to no fibrotic tissue was observed surrounding the cartilage scaffold (Fig. 5K), and this was confirmed with collagen type I and II staining (Fig. 5L). Staining for mouse MSC markers [Sca1 (Ly6A/E), CD140a (Pdgfra)] in DMSO (Fig. 5E-G) or drug 70 (Fig. 5M-O) demonstrates that these populations are present under both conditions. Because it is unlikely that tissue-resident MSCs can differentiate into the new cartilage tissue observed 3 days after injury with drug 70 treatment, the tissues were stained for Ki67 (Mki67) to identify whether and where proliferating cells were localized. In both DMSO- (Fig. 5H) and drug 70- (Fig. 5P) treated animals, a number of Ki67-positive cells are observed in the epidermal layers of the ear, with some positive cells observed in the connective tissue between the skin and cartilage layers. However, the staining within the cartilage scaffolds in DMSO- versus drug 70-treated animals was strikingly distinct. In DMSO-treated animals, the Ki67-positive cells were present in tissue that appeared to be fibrotic in nature (Fig. 5H), while in drug 70-treated animals, it appeared that the cartilage scaffold was positive for Ki67 (Fig. 5P). This was confirmed using staining for type I collagen and type II collagen (Fig. S6). To quantify this result, the area of tissue repair was harvested by applying a second 2 mm biopsy bunch to the injured area (thereby only removing the tissue that had ‘filled’ in the defect since the original injury and 3 days after injury), digesting this tissue and performing flow cytometry (Fig. 5Q,R). Significantly more type II collagen-positive cells were observed with drug 70 treatment, which validated the histology results, and overall more Ki67-positive (proliferating) cells were observed. Interestingly, fewer undifferentiated MSCs (Sca1^+^CD140a^+^) were observed with drug 70 treatment (Fig. 5Q). When we assayed for type II collagen-positive cells that were also positive for Ki67, we found that almost no type II collagen-positive cells were positive for Ki67 in DMSO-treated animals, but nearly 100% of the type II collagen-positive cells were Ki67 positive in drug 70-treated animals. Lastly, it was observed that the majority of the MSCs in DMSO- and drug 70-treated animals were positive for Ki67; however, there was a slight increase in Ki67-positive MSCs with drug 70 treatment (Fig. 5R).

#### p21^−/−^ mice

**Fig. 6.**
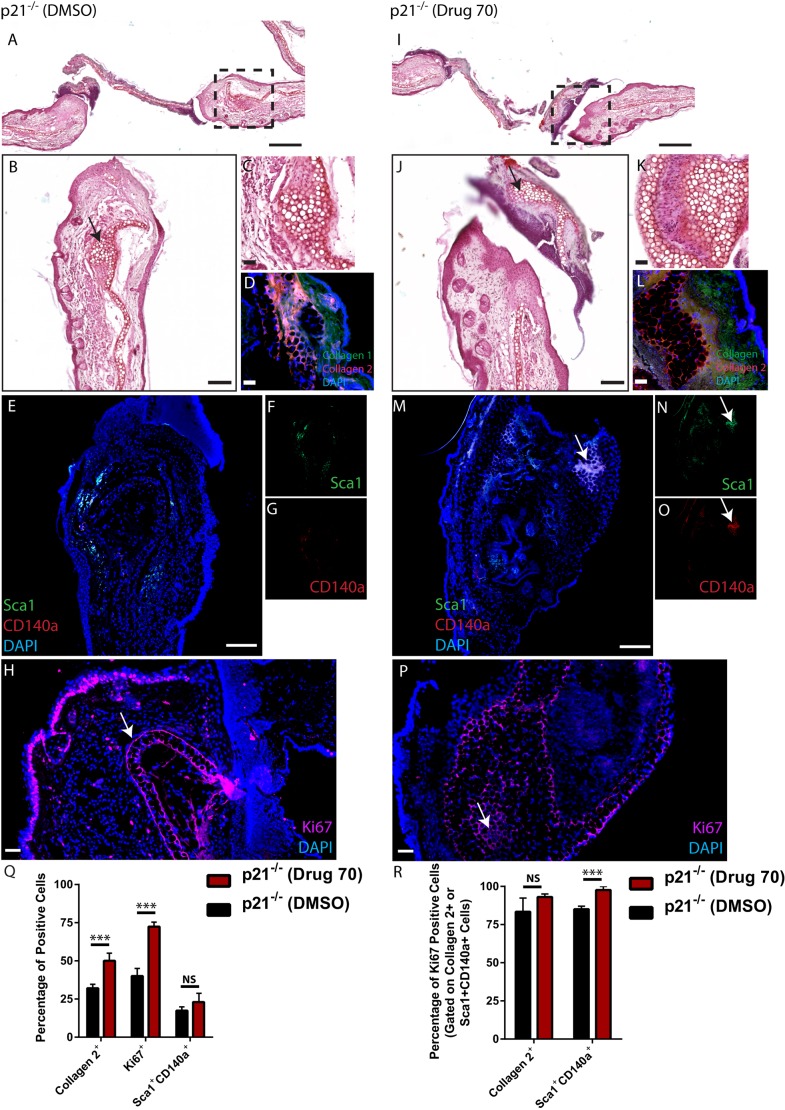
**Characterization of cells expressing MSC markers 3 days postinjury in p21^−/−^ mice.** (A,B) Injured p21^−/−^ mice treated with DMSO demonstrated robust cartilage present at the site of injury (arrow). (C,D) The cartilage scaffold was also positive for collagen type II. (E-G) Few cells expressing Sca1 (F), CD140a (G) or both (E) were present within the wound site. (I-L) With drug 70 treatment (I), new cartilage could be observed within the wound site (arrow, J) that was positive for Safranin O (K) and collagen type II (L). (M-O) Sca1 (N), CD140a (O) or double-positive cells (M) were present within the cartilage tissue (arrow). (H,P) In DMSO- (H) and drug 70- (P) treated animals, Ki67 was staining is present within the epidermis and cartilage scaffold of the ear (arrows). (Q,R) Flow analysis of the wound area in both DMSO- and drug 70-treated animals demonstrated that significantly more chondrocytes (collagen 2^+^) (Q) were present with drug 70 treatment, and that almost all chondrocytes were proliferating *in vivo* in both treatment groups (R). Data are mean±s.d.; NS, nonsignificant; ***P<0.01. Scale bars: 200 µm in A and I; 50 µm in B-D, H, J-L and P; 100 µm in E and M.

As observed in drug 70-treated C57BL/6 mice at 3 days postinjury, a large amount of tissue/wound infiltrate was observed in p21^−/−^ mice regardless of treatment group (Fig. 6A,B). This tissue also appeared to be transient/unstable as it was not seen at day 7 postinjury. p21^−/−^ mice demonstrated an expansion of the cartilage layer of the damaged ear at 3 days postinjury with DMSO treatment (Fig. 6A-C), which was positive for collagen type II staining (Fig. 6D). This was enhanced with drug treatment (Fig. 6I), with some areas of injured tissue being almost completely comprised of cartilage (Fig. 6J, arrow, K) which also stained positive for collagen type II (Fig. 6L). As in C57BL/6 mice, MSCs were present in both DMSO- (Fig. 6E-G) and drug 70-treated ears (Fig. 6M-O); however, in the drug 70-treated ears, cells positive for Sca1 and CD140a could be observed intermixed with a section of new cartilage tissue (Fig. 6M). Ki67 staining was observed within the epidermis layer, and also within the cartilage scaffold in p21^−/−^ mice treated with DMSO (Fig. 6H) or drug 70 (Fig. 6P). This was quantified using flow cytometry as performed in C57BL/6 mice. An increase in type II collagen-positive cells was observed in p21^−/−^ mice treated with drug 70, and a similar result was observed with total Ki67-positive cells (Fig. 6Q). No significant difference was observed in the number of Sca1^+^CD140a^+^ double-positive MSCs. Furthermore, no difference was found in the number of type II collagen-positive cells that were also positive for Ki67; however, it should be noted that in DMSO- and drug 70-treated p21^−/−^ mice, almost all chondrocytes were positive for Ki67 (Fig. 6R). An increase in the number of MSCs expressing Ki67 was observed in p21^−/−^ mice treated with drug 70 compared with those treated with DMSO (Fig. 6R).

### Effect of drug 70 on primary chondrocyte proliferation

**Fig. 7.**
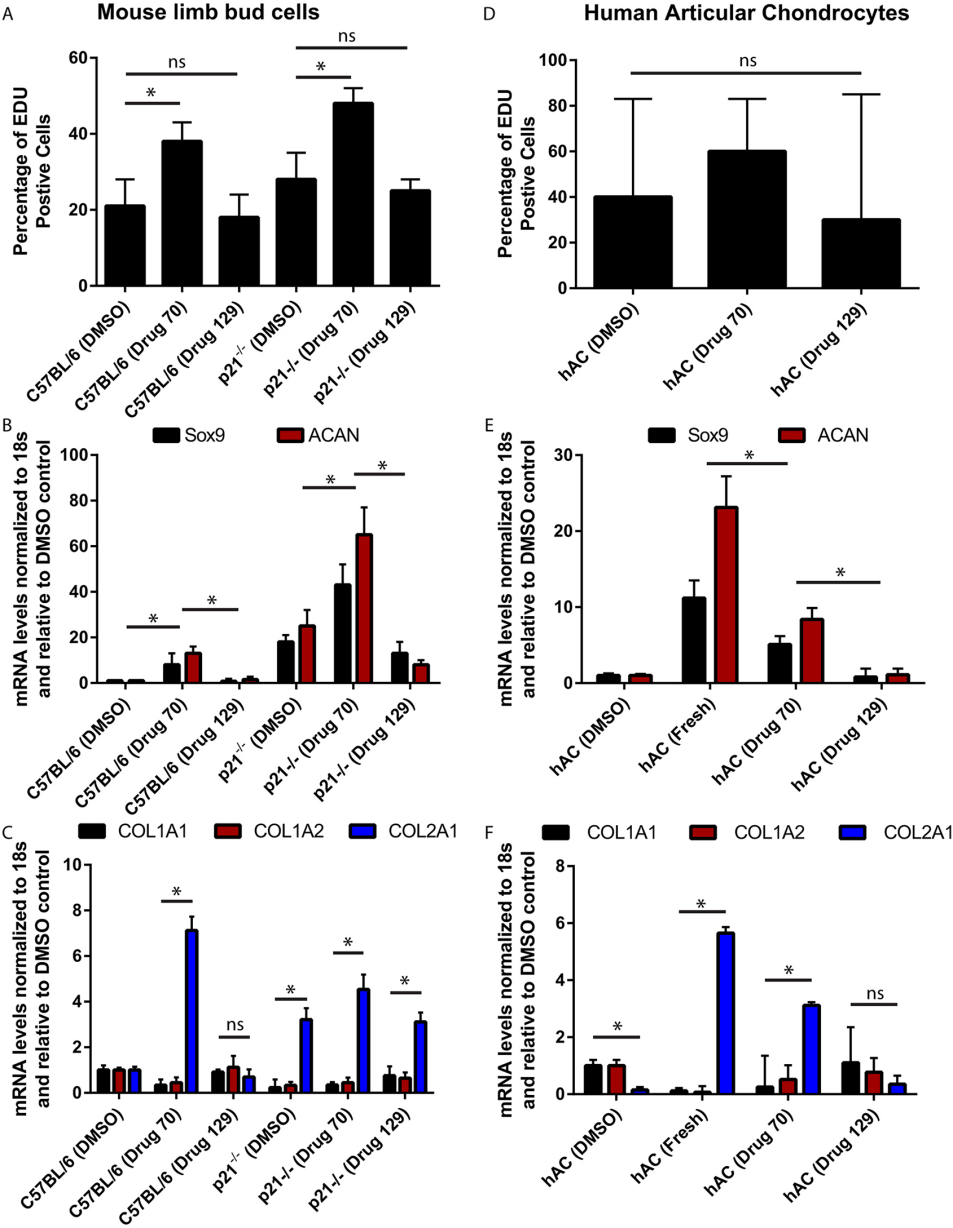
**Effect of drug 70 on chondrocyte proliferation and maintenance of the chondrogenic phenotype.** (A,B) Primary chondrocytes were obtained from embryonic limb buds of C57BL/6 and p21^−/−^ mice. They were treated with DMSO, drug 70 or an additional HSP90 inhibitor demonstrated not to affect p21 levels (drug 129) (A). Drug 70 induced proliferation in both C57BL/6 and p21^−/−^ chondrocytes, whereas DMSO and drug 129 had no effect (A). Maintenance of chondrogenic phenotype was assessed using the chondrogenic markers *Sox9* and *Acan*. Treatment of C57BL/6 and p21^−/−^ chondrocytes with drug 70 increased the expression of both markers, whereas drug 129 decreased *Acan* expression in p21^−/−^ chondrocytes. Overall, p21^−/−^ chondrocytes demonstrated increased marker expression than C57BL/6 chondrocytes regardless of treatment group (B). (C) It was also observed that drug 70, but not drug 129, treatment increased/maintained the expression of *Col2a1*, with a corresponding decrease in *Col1a1* and *Col1a2*. (D) In human articular chondrocytes (hAC), neither drug 70 nor drug 129 had any effect on proliferation in passaged chondrocytes. (E) However, drug 70 treatment maintained chondrocyte marker gene expression in passaged chondrocytes compared with fresh chondrocytes, whereas DMSO or drug 129 treatment did not. (F) A similar effect to that observed in mouse cells was observed in human chondrocytes, with drug 70 treatment increasing the expression of *COL2A1*, with a corresponding decrease in *COL1A1* and *COL1A2*. Data are mean±s.d.; ns, nonsignificant; *P<0.05.

Because it appeared from the histological analysis that chondrocytes (type II collagen^+^ cells) were proliferating *in vivo* and maintaining an auricular chondrocyte phenotype in response to drug 70 treatment, it was decided to directly test the ability of drug 70 to regulate chondrocyte proliferation and stabilization of the chondrogenic phenotype in mouse and human chondrocytes. Primary chondrocytes isolated from embryonic mouse limb buds were subjected to 0.1 µM drug 70 treatment for 24 h and assayed for proliferation using an EdU assay. In C57BL/6 chondrocytes treated with DMSO, ∼20% of the population had divided, while this was almost doubled in response to drug 70 treatment (Fig. 7A). In p21^−/−^ chondrocytes, ∼25% of the population had divided within 24 h and this was significantly increased with drug 70 treatment (Fig. 7A). To test whether this was due to more HSP90 inhibition versus p21 inhibition, a second HSP90 inhibitor, drug 129 (NVP-AUY922) (Nordin et al., 2015), was selected because it was also tested in the screen and demonstrated to have no effect on p21 expression levels. Drug 129 had no effect on chondrocyte proliferation in C57BL/6- or p21^−/−^-derived chondrocytes. As it has been established that chondrocytes in culture will de-differentiate and then begin to proliferate, we next examined whether drug 70 treatment was also able to maintain the chondrogenic phenotype while promoting proliferation. DMSO controls (C57BL/6 and p21^−/−^) demonstrated low levels of *Sox9* and *Acan* expression, but these mRNAs were increased in the presence of drug 70 (Fig. 7B). In general, p21^−/−^ chondrocytes demonstrated higher levels of both markers than cells derived from C57BL/6 animals regardless of drug 70 treatment. Whereas drug 129 had no effect on chondrogenic markers in C57BL/6 chondrocytes, it significantly decreased *Acan* expression in p21^−/−^ cells (Fig. 7B). We also examined the expression of collagen type 1 (*Col1a1* and *Col1a2*) and collagen type 2 (*Col2a1*) to determine whether drug treatment altered collagen expression (Fig. 7C). Drug 70 treatment of C57BL/6 and p21^−/−^ cells resulted in an increase in type 2 expression, with a corresponding decrease in type 1 expression; drug 129 had no effect on collagen (type 1 or 2) expression. Because mouse limb bud chondrocytes can represent a mixed population of cells with differential chondrogenic potential/commitment, it was also decided to test the effect of drug 70 on human articular chondrocytes in culture. Although no difference in the percentage of EdU-positive cells was observed in response to drug 70 or drug 129 treatment (Fig. 7D), a partial maintenance of chondrogenic gene expression was observed when chondrocytes were passaged in the presence of drug 70, compared with freshly isolated chondrocytes. This effect was not observed with drug 129 treatment (Fig. 7E). Similar to what was observed in the mouse cells, drug 70 treatment and not drug 129 treatment induced/maintained the expression of *COL2A1*, with a corresponding decrease in *COL1A1* and *COL1A2* expression (Fig. 7F).

### Contribution of macrophage populations

**Fig. 8.**
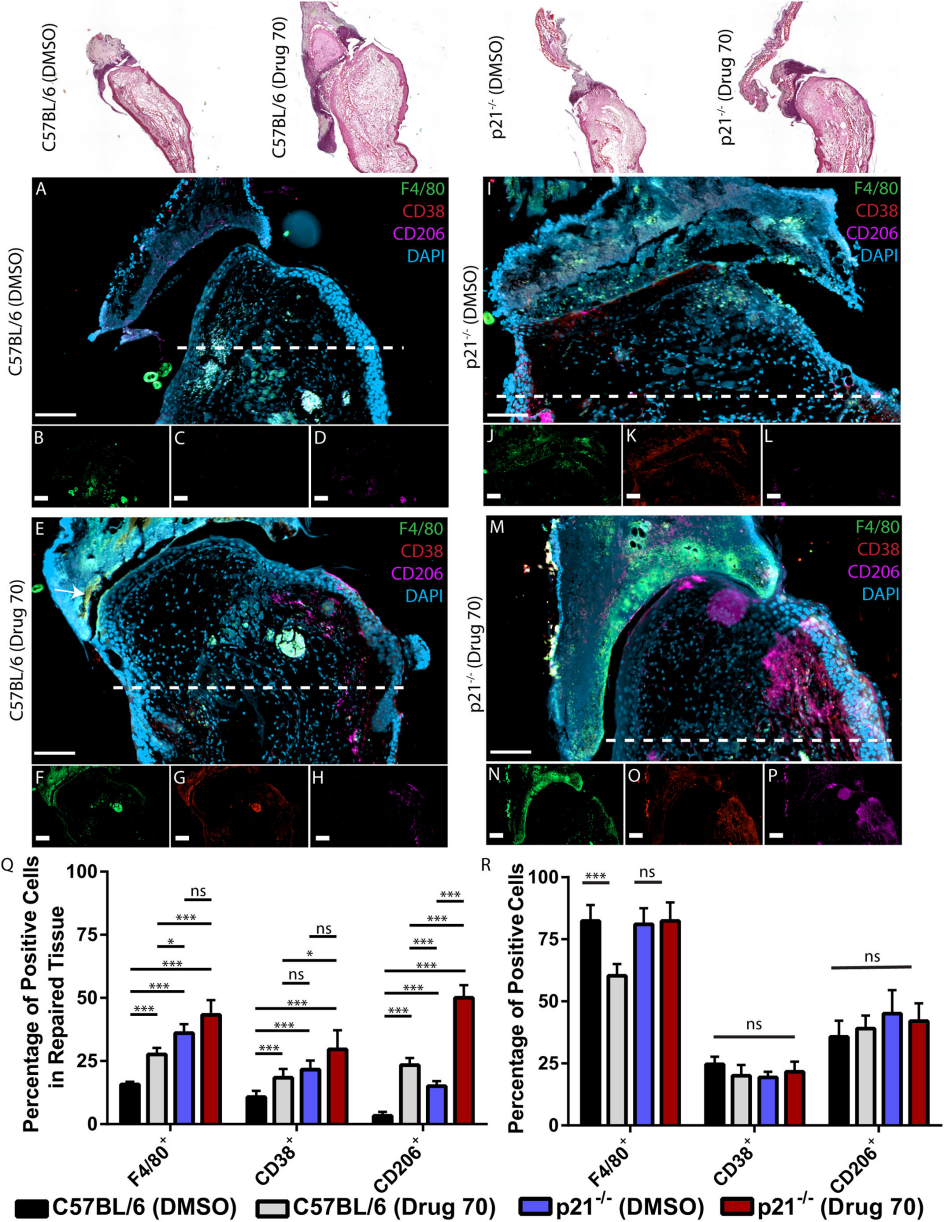
**Characterization of cells expressing** **macrophage markers 3 days postinjury in C57BL/6 and p21^−/−^ mice.** Top row: Representative histological images of the different mouse strains and treatment groups. (A-D) Injured C57BL/6 mice treated with DMSO (A) display F4/80-positive cells in the wound area (B), but minimal CD38- (C) and CD206- (D) positive cells are observed. (E-H) With drug 70 treatment (E), F4/80-positive cells are observed (F) and increased CD38 (G) and CD206 (H) staining is present. (I-L) In p21^−/−^ mice treated with DMSO (I), robust F4/80 (J) and CD38 (K) staining is observed, and CD206 is detectable (L). In p21^−/−^ mice treated with drug 70 (M), robust F4/80 (N), CD38 (O) and CD206 (P) staining is present within the wound site. (Q) Flow analysis of the wound area in both DMSO- and drug 70-treated animals (C57BL/6 and p21^−/−^ mice) demonstrates that significantly more total^(F4/80+)^, M1^(CD38+)^ and M2^(CD206+)^ macrophages are present in the wound site in drug 70-treated animals versus DMSO controls. (R) Drug 70 treatment inhibits the differentiation of monocytes to macrophages in C57BL/6, but not p21^−/−^, mice. (R) Drug 70 treatment has no effect on the polarization of macrophages from C57BL/6 or p21^−/−^ mice. Data are mean±s.d.; ns, nonsignificant; *P<0.05, ***P<0.01. Scale bars=50 µm.

Recently, Simkin et al. (2017) demonstrated that macrophage populations are essential for ear wound regeneration in Spiny mice. Therefore, to determine whether drug 70 treatment might also be acting through regulation of macrophages and/or polarization of macrophages, we assayed for total macrophages (F4/80; Adgre1), M1 (CD38) and M2 (CD206; Mrc1) in DMSO- and drug 70-treated animals. In injured C57BL/6 mice treated with DMSO (Fig. 8A), F4/80-positive cells were observed adjacent to the wound site (Fig. 8B), but few M1 or M2 macrophages were observed (Fig. 8C,D). Following drug 70 treatment, cells expressing F4/80, CD38 or CD206 were all present in the wound area (Fig. 8E-H), but none appeared to be directly associated with the new cartilage. In p21^−/−^ mice treated with DMSO, although all markers were present, primarily, M1^(F4/80+CD38+)^ macrophages were localized to areas of new cartilage formation (Fig. 8I-L). In p21^−/−^ mice treated with drug 70, we observed that M1 and M2^(F4/80+CD206+)^ cells were both localized to areas of new cartilage formation (Fig. 8M-P). These results were corroborated by flow cytometry analysis of the macrophage populations in the wound tissue (Fig. 8Q). To determine whether drug 70 treatment augmented macrophage differentiation and/or polarization, mouse monocytes were isolated, differentiated and polarized in the presence/absence of drug 70. A significant decrease in the differentiation of monocytes to macrophages was observed in C57BL/6 (but not p21^−/−^ mice) in the presence of drug 70 (Fig. 8R). No differences were observed in the polarization of macrophages to M1 or M2 subtypes in any treatment group or mouse strain.

## DISCUSSION

Although cartilage regeneration is not readily observed in mammals, it occurs naturally in urodeles, such as newts and salamanders (Vlaskalin et al., 2004). When a limb is removed, a mesenchymal growth zone, or blastema, is formed locally, and this tissue mass has the plasticity to differentiate into articular cartilage (Echeverri et al., 2001). As mentioned earlier, both MRL and p21^−/−^ mice have the ability to regenerate cartilage after injury and it was previously demonstrated that MRL mice display comparatively low levels of p21 (Bedelbaeva et al., 2010). This strongly suggests that p21 plays a role in the suppression of cartilage regeneration and, therefore, that modulation of p21 expression might be a viable therapeutic approach for inducing endogenous cartilage repair. In the context of this project, we undertook a small molecule inhibitor screen to identify candidate drugs that modify p21 expression at the transcriptional level in order to mimic the effects of p21 loss/knockdown. Although p21 is primarily known as a cell cycle inhibitor, regulating downstream of p53, the observation that p53^−/−^ mice do not exhibit a regenerative phenotype (Arthur et al., 2010) suggests that p21 might be playing an alternative role from a p53 (Trp53)-mediated pathway in terms of chondrogenic differentiation/repair. None of the compounds identified in the screen had been previously shown to inhibit p21, yet they are known to block target pathways upstream of p21. The drugs identified and chosen for further *in vitro* analysis in the current study included an HSP90 inhibitor (drug 70), HDAC/HER2 (Erbb2)/EGFR multi-inhibitor (drug 93), two mTOR inhibitors (drugs 102 and 107), and a PIK3/mTOR inhibitor (drug 111). Interestingly, all the drugs tested in this study not only reduced the level of *p21* mRNA expression, but all induced some level of chondrogenesis in human synovial MSCs, suggesting that at the concentrations used, the effect of inhibiting p21 was conserved in terms of chondrogenesis. However, off-target results and/or pathway ‘crosstalk’ cannot be discounted based on the experimental design we employed. It is critical to note that not all drugs tested were able to reduce p21 protein levels. This could be due to the half-life of p21 (between 1 h and 6 h in HCT-116 cells, unknown in primary synovial MSCs) and the time points examined, but could also be caused by the large differences in p21 protein levels between normal and OA MSCs. We previously demonstrated that basal levels of p21 expression (mRNA and protein) are distinct between normal and OA cells (Masson et al., 2015), with normal cells demonstrating significantly less expression of p21.

Drug 70, the HSP90 inhibitor, was further examined *in vivo* owing to its capacity to inhibit p21 (at mRNA and protein levels), induce proliferation, enhance accumulation of cells in the G2/M phase and induce chondrogenesis. Although other agents demonstrated many of these effects, drug 70 was the only candidate to meet all of these criteria. While we cannot conclusively distinguish between the effects of drug 70 on p21 inhibition versus HSP90 inhibition, it should be noted that the other HSP90 inhibitor in the study (NVP-AUY922) was not able to induce the proliferation of chondrocytes or maintain a chondrogenic phenotype in these primary cells. However, we know that the effects observed with drug 70 were not solely due to p21 inhibition, as there was synergy observed in the ectopic formation of cartilage, macrophage recruitment/polarization and *in vivo*/*in vitro* chondrocyte proliferation in p21^−/−^ mice with drug 70 treatment. This suggests that drug 70 is not solely acting through p21 and that HSP90 inhibition itself might also play a role in chondrogenesis as previously suggested (Loones and Morange, 1998). Although the literature on HSP90 inhibition and chondrogenesis is minimal, a recent study has demonstrated that oral administration of the HSP90 inhibitor BIIB021 protected against biomechanically induced OA in rats (Siebelt et al., 2013).

Macrophages have been intimately implicated in the onset and progression of OA as they are known to produce (or initiate production of) many of the pro-inflammatory cytokines that drive the disease process (de Lange-Brokaar et al., 2012). A recent study by Simkin et al. (2017) identified that spontaneously healing mice not only demonstrate a more robust immune/macrophage response after healing, but that macrophages are required for the ability to self-regenerate tissue in these models. Although there appears to be discrepancy between macrophages driving disease progression in the joint versus requirement for tissue (e.g. cartilage) repair in the ear, a recent study by Wu et al. (2017) demonstrated that macrophage depletion in obese injured mice does not attenuate OA progression/severity. This suggests that more insight is required into the role of macrophages in OA/cartilage degeneration. However, in terms of our current study, it was observed that drug 70 treatment of injured ears correlated with increased recruitment of macrophages (M0, M1 and M2 polarization states), similar to what was observed in p21^−/−^ mice; however, a synergy between the absence of p21 and drug 70 treatment on macrophage recruitment demonstrates that this effect is not only due to p21, but potentially HSP90 (or an off-target pathway) inhibition. There is evidence in the literature linking HSP90 to both macrophage recruitment and behavior (Bzowska et al., 2017), so this would warrant additional investigation in our ear injury model.

Instead of targeting the processes that are degrading cartilage, there have been attempts made to promote new cartilage growth, similar to this project (Hunter et al., 2010; Lohmander et al., 2014). One promising substance for stimulating chondrogenic differentiation is kartogenin (KGN). This small molecule was identified from a screen to induce chondrogenesis in human MSCs (Johnson et al., 2012). Although this compound is not yet in human clinical trials, a study was recently published showing promising results of KGN treatment in a rabbit model of OA (Shi et al., 2016), in which new hyaline-like cartilage was formed where the defect was induced. Interestingly, KGN regulates the RUNX family of transcription factors (Johnson et al., 2012) and p21 is repressed transcriptionally by RUNX1 (Lutterbach et al., 2000). As it remains unknown if KGN inhibits p21 transcription, it would be of interest to determine whether this is a potential mode of action for the success observed with KGN in regulating chondrogenic differentiation *in vivo* and *in vitro*.

It is also important to note that in the current study we were not able to test the effect of drug 70/17-DMAG in an articular cartilage injury/OA model. These experiments are crucial because auricular and articular cartilage exist in different environments and experience biomechanical forces. We therefore cannot generalize our results on auricular cartilage repair/regeneration to articular cartilage and are in the process of undertaking these experiments.

In conclusion, we have identified five putative p21 inhibitors (at the expression level). All inhibitors tested had the ability to induce chondrogenesis in human synovial MSCs derived from normal individuals and patients with OA. Drug 70 (17-DMAG) was selected for further *in vivo* study and was found to enhance auricular repair through two potential mechanisms, the first being an induction of proliferation of chondrocytes (with maintenance of chondrogenic phenotype) and the second being enhanced recruitment of macrophages to the injury site. Although more research is required to determine the exact mechanism by which drug 70/17-DMAG is inducing cartilage formation *in vivo*, based on the human and mouse data in this paper, we suggest that 17-DMAG could be a novel therapeutic for cartilage regeneration worthy of additional study.

## MATERIALS AND METHODS

### Human ethics statement

This study protocol was approved by the University of Calgary Human Research Ethics Board (REB15-0005 and REB15-0880). For the normal group (*n*=5), criteria for control cadaveric donations were an age of 18 years or higher; no history of arthritis, joint injury or surgery (including visual inspection of the cartilage surfaces during recovery); no prescription anti-inflammatory medications; no co-morbidities (such as diabetes/cancer); and availability within 4 h of death (Masson et al., 2015; O'Brien et al., 2017). For the knee OA group (*n*=7), inclusion criteria were based on a diagnosis of OA performed by an orthopedic surgeon at the University of Calgary, based on clinical symptoms with radiographic evidence of changes associated with OA in accordance with the American College of Rheumatology (ACR) criteria. Radiographic evidence of OA based on a K/L grading of 3 or 4 was required to be included in the study. Patient demographics are presented in Table 1.

**Table 1.**
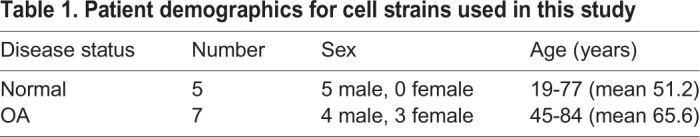
**Patient demographics for cell strains used in this study**

### Animal ethics statement

Animal studies were carried out in accordance with the recommendations in the Canadian Council on Animal Care guidelines. Animal protocols (AC16-0043) and surgical procedures in this study were approved by the University of Calgary Health Sciences Animal Care Committee.

### Cell lines

#### Synovial MSCs

Normal synovial MSCs were derived and purified from healthy knee synovium tissue by the Southern Alberta Tissue Donation Program. OA synovial MSCs were derived and purified from the tissue of consenting patients undergoing a total knee joint replacement from Dr James Powell (University of Calgary, Department of Surgery, Calgary, Canada). The biopsies were minced and placed into Dulbecco's modified Eagle medium (DMEM):F12 medium with human MSC stimulatory supplement (StemCell Technologies, Vancouver, Canada). At 7-14 days after initial seeding (∼passage 2), the cells were processed for magnetic purification using the Human Lineage Cell Depletion Cocktail (BD Canada, Ontario, Canada) that removed CD3-, CD14-, CD16-, CD19-, CD41a-, CD56- and glycophorin A-positive cells. This cell population was then selected for the CD90^+^ (BD) progenitor fraction using magnetic separation (Harris et al., 2013; Masson et al., 2015). Marker profile expression (CD90^+^, CD105^+^, CD73^+^, CD44^+^, CD45^−^, CD11b^−^) was confirmed for each cell line employing standard flow cytometry practices. In addition, each cell line was tested for differentiation potential for three lineages (bone, cartilage, fat) (Harris et al., 2013).

#### XMAN™ p21 reporter cell line

The XMAN™ NanoLuc™-PEST p21 promoter reporter cell line was acquired from Horizon Discovery (Waterbeach, UK). In this cell line, NanoLuc™-PEST is placed directly downstream of the *CDKN1A* endogenous start codon in a parental HCT116 cell line. The activity of the endogenous promoter can be assessed through luciferase. Cells were grown in DMEM:F12 medium containing 10% fetal bovine serum (FBS), 1% nonessential amino acids, 1% penicillin-streptomycin and 0.1% beta-mercaptoethanol (all Thermo Fisher Scientific, Carlsbad, CA, USA). XMAN™ cells were passaged by trypsinization at a ratio of 1:10 owing to their high proliferation rate.

### Compound screening

XMAN™ cells were plated at 50,000 cells per well in Grenier white 96-well plates, allowed to adhere overnight, treated with 10, 1, 0.1 and 0.01 µM of each drug in duplicate for 24 h. The XMAN™ NanoLuc™ Reporter Kit was then used to activate the luminescence. Briefly, Nano-Glo™ Luciferase Assay Reagent was prepared by combining one volume of Nano-Glo™ Luciferase Assay Substrate to 50 volumes of Nano-Glo™ Luciferase Assay Buffer (Horizon Discovery). One volume of the Nano-Glo™ Luciferase Assay Reagent equal to the volume of media on the cells was then added directly to each well and mixed. After 3 min, luminescence was quantified (Victor X3 plate reader) and analyzed against a carrier control (DMSO), and concentration- and plate-specific controls.

### Drug library

The drug library used contained 146 small molecule inhibitors in various stages of preclinical and clinical development (Tables S1-S4) (McKenzie et al., 2015). The compounds are agents provided by pharmaceutical companies after review of the study proposal and legally approved material transfer agreement and agents purchased from medicinal chemists. All therapeutic agents used in the screening analysis were synthesized, checked for purity, and provided by Chemietek (Indianapolis, IL, USA).

### Drug-induced metabolic toxicity

AlamarBlue (Thermo Fisher Scientific) was used to detect the metabolic activity of the cells (both XMAN™ NanoLuc™ and human synovial MSCs) after exposure to selected drugs at the same four concentrations (0.01, 0.1, 1 and 10 µM). Briefly, AlamarBlue was added after 24 h of drug treatment and incubated for 4 h in the dark at 37°C. The absorption was quantified using a Benchmark Plus microplate spectrometer at 570 nm using 600 nm as a reference wavelength, and analyzed against a plate-specific carrier control (DMSO), as well as a medium only control.

### Analysis of cell proliferation

A Click-iT^®^ EdU Flow Cytometry Assay Kit (Thermo Fisher Scientific) was used to measure the percentage of cells that passed through the S-phase over 24 h of drug condition treatment. Human synovial MSCs were plated at 50,000 cells per well in six-well plates for each condition, allowed to adhere overnight and treated with the respective condition. Cells were labeled with 10 µM EdU during the first 24 h of treatment, then washed with 1% bovine serum albumin (BSA) in PBS. Cells were treated with 100 µl Click-iT^®^ fixative, then washed with 1% BSA in PBS, and mixed with 100 µl 1× Click-iT^®^ saponin-based permeabilization and wash reagent. Click-iT^®^ reaction cocktail was added to each tube and incubated. Cells were then washed and assayed for EdU by flow cytometry. Flow cytometry was performed on an Attune™ Flow Cytometer. At least 10,000 gated events (excluding debris) were counted at a wavelength of 488 nm.

### Propidium iodide analysis of the cell cycle

Human synovial MSCs were plated at 50,000 cells per well in six-well plates for each condition, allowed to adhere overnight and treated with the respective condition. After treatment, the cells were fixed by suspending in ice-cold PBS and vortexed gently, while cold 70% ETOH was added dropwise to prevent clumping. The solution was stored overnight at 4°C. Cells were then washed twice with PBS, pelleted and stained with 50 µl 100 µg/ml ribonuclease (Sigma-Aldrich, St Louis, MO, USA) and 200 µl 50 µg/ml propidium iodide (Sigma-Aldrich), and incubated for 30 min at room temperature. Samples were then run on an Attune™ Flow Cytometer and analyzed using Attune™ software.

### *p21* mRNA (qPCR)

Human synovial MSCs were plated at 5000 cells per well in 96-well dishes and allowed to adhere overnight. Cells were treated with each drug condition for 24, 4, 1 and 0.5 h. Cells were trypsinized and pelleted. The RNA from each sample was extracted using EZNA^®^ Total RNA Kit I (Omega, Norcross, GA, USA). RNA was converted to cDNA using a High Capacity Reverse Transcriptase cDNA kit (Thermo Fisher Scientific). mRNA levels were analyzed using a TaqMan^®^ Universal PCR Master Mix using TaqMan^®^ Gene Expression Assay primers for human *CDNK1A* (*p21*) and *18S* (endogenous control) on a 7900HT Fast-Real-Time PCR System (all Thermo Fisher Scientific). Samples were run in triplicate and analyzed against a carrier control (DMSO) using GraphPad Prism 6.

### Chondrogenesis

Human synovial MSCs were pelleted at 50,000 cells per pellet in 1.5 ml Eppendorf tubes by centrifugation. Cells were cultured in DMEM:F12 medium with 10% FBS, 1% Anti-Anti and 1% nonessential amino acids (all Thermo Fisher Scientific), as well as the respective drug condition for 21 days. Chondrogenic control (chondro) was cultured in medium containing 500 ng/ml BMP-2 (Peprotech, Rocky Hill, NJ, USA), 10 ng/ml TGF-β3 (Peprotech), 10 M dexamethasone, 50 μg/ml ascorbic acid, 40 μg/ml proline and 100 μg/ml pyruvate, and supplemented with 1× insulin, transferrin and selenium (all Sigma-Aldrich), and the pH of the final solution was adjusted to 7 with NaOH. Undifferentiated control was pelleted and cultured in medium without any growth factors or drugs. Cell pellets were used for histology and mRNA analysis as well as carrier and medium only controls. The medium was changed every 2-3 days, with special attention not to disturb the pellet.

### qPCR for analysis of chondrogenesis

Pellets were lysed in TRIzol (Ambion) with an 18- or 21-gauge needle, then total RNA was extracted using EZNA^®^ Total RNA Kit I (Omega). Briefly, 200 µl chloroform per milliliter of TRIzol was added to the TRIzol solution. Samples were vortexed, incubated at room temperature for 15 min, then centrifuged for 15 min at 4°C and 12,000 ***g***. The top, clear, layer was transferred into a new HiBind^®^ RNA mini column in a 2 ml collection tube. The sample was centrifuged at 10,000 ***g*** for 1 min, and the filtrate was discarded. The sample was washed with 500 µl wash buffer I, centrifuged for 30 s at 10,000 ***g*** and the filtrate was discarded. The sample was washed with 500 µl wash buffer II, centrifuged for 1 min at 10,000 ***g*** and the filtrate was discarded. The sample was then centrifuged at 14,000 ***g*** for 2 min to remove any excess liquid. The collection tube was replaced with a new one, and the sample was extracted by adding 50 µl ultra-pure water (Thermo Fisher Scientific) and centrifuging at 14,000 ***g*** for 2 min. The sample was immediately stored at −80°C until the next step could be completed. RNA (5 µg) was converted to cDNA using a High Capacity Reverse Transcriptase cDNA kit. mRNA levels were then analyzed using TaqMan^®^ Universal PCR Master Mix using TaqMan^®^ Gene Expression Assay primers for human *CDKN1A* (*p21*), *SOX9*, *ACAN*, *COL2A1*, *COL1A1*, *COL1A2* and *18S* (endogenous control) (all Thermo Fisher Scientific) on a 7900HT Fast-Real-Time PCR System. Samples were run in triplicate and resulting threshold (Ct) values were analyzed using the ΔΔCt method against 18S endogenous control and DMSO as the reference untreated control. The relative mRNA expression was plotted using GraphPad Prism 6.0. Statistical analysis was performed on the ΔΔCt values, as the relative mRNA expression values are log transformed.

### Pellet size

Pellets were fixed overnight at 4°C in 4% paraformaldehyde (BDH) and then washed three times with PBS. After fixation, photos were taken using Axio Zoom^®^ (Zeiss) of each pellet. The area of the pellet was determined for each using ImageJ software (https://imagej.nih.gov/ij/) and analyzed using GraphPad Prism 6.

### *In vivo* mouse wound healing model

Ten-week-old C57Bl/6 mice (*n*=32) and p21^−/−^ mice (*n*=26) were used for the ear wound healing experiments. Mice were split into five treatment groups: C57Bl/6 low dose (*n*=16), C57Bl/6 high dose (*n*=4), C57Bl/6 control (*n*=12), p21^−/−^ low dose (*n*=16) and p21 control (*n*=11). All mice were placed under isoflurane anesthesia and a 2 mm through-and-through ear punch was given to the center of the left ear at week 0. Mice in the drug group were given topical treatment of low dose (0.1 µM), high dose (100 µM) or control (0 µM) drug 70 in DMSO gel (Life Choice, Alberta, Canada) adjacent to the circular wound. Mice were treated with the respective condition once per week and sacrificed at 3, 7, 14 or 28 days postinjury. Images of the wound were taken with a size standard in frame once per week for 4 weeks. Images were analyzed for wound diameter using ImageJ software.

### Histology

Ears were harvested and fixed in neutral buffered formalin (Sigma-Aldrich) for 48 h. Samples then underwent tissue processing using a Leica TP1020 automatic tissue processing machine. Tissue was then embedded in paraffin wax blocks and sections were cut with a Leica Automated Rotary Microtome (RM 2255) into 10 µm sections and transferred onto slides. The slides were stained with Safranin O and Fast Green and imaged using an Axio Scan (Zeiss) at 10× magnification. Images were compiled and analyzed with Zeiss Zen software.

### Immunofluorescence

Tissue sections on slides were also processed for immunofluorescence. Primary antibodies conjugated to fluorophores included anti-Sca1 (clone D7), anti-CD140a (clone APA5) (MSC markers), anti-F4/80 (clone BM8), anti-CD38 (clone 90), anti-CD206 (clone MR6F3) (macrophage), anti-Ki67 (clone SolA15) (proliferation) (all Thermo Fisher Scientific), and anti-collagen I (clone 8-3A5) and II (clone CIIC1) (Developmental Studies Hybridoma Bank, University of Iowa, Iowa City, IA, USA). All slides were counterstained with the nucleic acid stain 4′,6-diamidino-2-phenylindole (DAPI) (Sigma-Aldrich) and mounted using FluorSave reagent (Calbiochem, Darmstadt, Germany). Isotype controls for Alexa Fluor 488, 568 or 647 demonstrated little to no reactivity. Slides were imaged using a Plan-Apochromat objective (20×20×/0.8 M27) on an Axio Scan.Z1 Slide Scanner microscope (Carl Zeiss, Oberkochen, Germany).

### Flow cytometry

To quantify cells present within the wound site, the mice were re-injured with a 2 mm through-and-through ear punch in the same area as the initial ear wound injury. This allowed us to collect the tissue deposited from the initial time of injury. The ear tissue was dissociated using the gentleMACS™ Dissociator (Milteny, Bergisch Gladbach, Germany) according to the manufacturer’s procedure. The resultant cell suspension was filtered and resuspended in 500 μl 90% MeOH and left for 5-10 min at room temperature. The cells were then centrifuged, the liquid was removed and 500 μl 0.1% Tween 20 was added to permeabilize the cells for 20 min at room temperature. The cells were centrifuged again, the liquid was removed, and 50 μl Tween buffer and 0.5 μg antibody (same antibodies as described in the ‘Immunofluorescence’ section) were added to each tube and incubated in the dark for 30-45 min at room temperature. The cells were then washed three times with FACS buffer, resuspended in FACS buffer and measured using FACS Caliber. The results were analyzed using FlowJo software (FlowJo, LLC).

### Primary chondrocyte analysis

Mouse embryos at embryonic day (E) 11.5 to E12.5 were collected as described previously (Underhill et al., 2014). The limb buds were carefully removed from the body wall with forceps. The limb bud was minced and transferred into Puck's saline A (PSA, Thermo Fisher Scientific) solution in a sterile 15 ml centrifuge tube and centrifuged for 5 min at 200 ***g***. The supernatant was removed and the limbs were re-suspended in 3 ml limb bud dissociation medium (PSA supplemented with 10% FBS and containing 1 U/ml dispase; Thermo Fisher Scientific). The tubes were incubated for 60 min at 37°C with gentle agitation, and gently vortexed every 20 min. The resultant cell suspension was strained through a 40 μm strainer. The cells were then plated in 40% DMEM high-glucose, 60% F12 medium supplemented with 10% FBS with penicillin, streptomycin and L-glutamine.

Human normal articular cartilage (tibia plateau) samples (*n*=3; two males/one female, mean age 54.2±5.3 years) were obtained from the Southern Alberta Tissue Donation Program. Cartilage tissue was cut into pieces of ∼2 mm^2^, then incubated with 1 mg/ml pronase (Thermo Fisher Scientific) for 30 min at 37°C (100 rpm). The cartilage was then incubated with 1 mg/ml collagenase (Sigma-Aldrich) for 24 h at 37°C (100 rpm). The resultant suspension was filtered (70 μm) and centrifuged. The chondrocytes were resuspended in DMEM:F12 medium supplemented with 10% FBS and Anti-Anti (all Thermo Fisher Scientific).

### Monocyte isolation and macrophage differentiation

Murine monocytes were isolated from C57 and p21^−/−^ mice using the EasySep™ Mouse Monocyte Isolation Kit (StemCell Technologies), following the manufacturer’s protocol. Isolated cells were incubated in DMEM supplemented with 10% heat-inactivated FBS, Anti-Anti and macrophage colony stimulating factor (all Thermo Fisher Scientific). Cells were classically activated (M1) with lipopolysaccharide (100 ng/ml, Sigma-Aldrich)+IFN-γ (20 ng/ml, Peprotech), or alternatively activated (M2) with IL-4 (20 ng/ml, Peprotech). All conditions were performed in the presence/absence of drug 70. Cells were harvested 24 h poststimulation and assayed by flow cytometry using antibodies against F4/80 (pan-macrophage), CD38 (M1) or CD204 (M2).

### Statistics

Statistical analysis of AlamarBlue, EdU, propidium iodide and mouse ear wound healing data was performed using standard one-way ANOVA with a Holm–Sidak multiple comparisons test, to determine significance relative to the treatment group relative to the DMSO carrier control group. The *P*-value cutoff was set at 0.05. RT-qPCR (both for p21 expression and following chondrogenesis) was analyzed with a standard two-way ANOVA for each drug condition with a Dunnett multiple comparisons test to determine the significance relative to the DMSO carrier control. Analysis of RT-qPCR was performed using the ΔΔCt values rather than the plotted logarithmically transformed R values, as a normally distributed data set is required for this analysis. The *P*-value cutoff was set at 0.05 for these tests as well. All statistics were performed using GraphPad Prism 6 software.

## Supplementary Material

Supplementary information
