# Supplementary material for: 17-DMAG regulates p21 expression to induce chondrogenesis in vitro and in vivo
Source: Dis Model Mech. 2018 Oct 8;11(10):dmm033662. doi: 10.1242/dmm.033662 (PMC6215425; doi:10.1242/dmm.033662)
Supplement: Supplementary information [file dmm-11-033662-s1.pdf]

Number	Inhibitor	Activity
1	CP-690550	JAK3 inhibitor
2	Docetaxel	Tubulin stabilizer
3	TGX-221	PI3K inhibitor
4	Lapatinib	HER1/2, EGFR inhibitor
5	PD 0325901	MEK inhibitor
6	PIK-75, Hydrochloride	PI3K inhibitor
7	ABT-263	Bcl-2 inhibitor
8	Axitinib (AG-013736)	VEGF-R inhibitor
9	AZD05030 (Saracatinib)	Src inhibitor
10	Canertinib (CI-1033)	ErbB-R inhibitor
11	GDC-0941	PI3K inhibitor
12	Bosutinib (SKI-606)	Abl, Src inhibitor
13	Nilotinib	Bcr-Abl inhibitor
14	FTY720, Hydrochloride	Immunosuppressant
15	ABT-888 (Veliparib)	PARP inhibitor
16	BIBW 2992 (Tovok)	RTK inhibitor
17	GDC-0449	Hedgehog pathway inhibitor
18	Vandetanib (Zactima)	RTK inhibitor
19	Vatalanib Dihydrochloride	RTK inhibitor
20	AZD 2281 (Olaparib)	PARP inhibitor
21	Bicalutamide (Casodex)	Androgen receptor inhibitor
22	BI 2536	PLK inhibitor
23	ZM 447439	Aurora kinase inhibitor
24	Pp242	mTOR inhibitor
25	OSI-906	IGF-1R inhibitor
26	LBH-589 (Panobinostat)	HDAC inhibitor
27	Laropiprant	PGD2-R antagonist
28	MK-2206	Akt inhibitor
29	Ramatroban (Bay u3405)	CRTH2-R antagonist
30	BMS-599626	RTK inhibitor
31	BMS-754807	IGF-1R inhibitor
32	Raltegravir	HIV integrase inhibitor
33	RDEA119	MEK inhibitor

Table S1. Small Molecule Compounds 1-33. Drugs screened within the current study including common name and mode of action.

Disease Models & Mechanisms • Supplementary information

Number	Inhibitor	Activity
34	PF-2341066	c-MET inhibitor
35	AZD 6244 (ARRY-142886)	MEK inhibitor
36	Odanacatib (MK-0822)	Cathepsin inhibitor
37	MS-275	HDAC inhibitor
38	NVP-TAE684	NPM-ALK inhibitor
39	SN-38	Topoisomerase I inhibitor
40	VX702	p38 MAPK inhibitor
41	MGCD0103	HDAC inhibitor
42	Maraviroc (UK-427857)	CCR5 antagonist
43	VX-680	Aurora kinase inhibitor
44	AN2728	PDE4 inhibitor
45	Bexarotene (Targretin)	RXR activator
46	Capecitabine (Xeloda)	NA synthesis inhibitor
47	CVT-6883	Adenosine receptor antagonist
48	Motesanib (AMG-706)	RTK inhibitor
49	Imatinib	TK inhibitor
50	Hypothemycin	T cell activation inhibitor
51	Dimebolin Hydrochloride	Antihistamine
52	FK-506	Immunosuppressant
53	Vorinostat (SAHA)	HDAC inhibitor
54	Dasatinib	Src inhibitor
55	Montelukast Sodium (Singulair)	LTR antagonist
56	Rofecoxib (Vioxx)	COX-2 inhibitor
57	Pemetrexed Disodium (Alimta)	NA synthesis inhibitor
58	Gemcitabine, HCl (Gemzar)	NA synthesis inhibitor
59	Doxorubicin (Adriamycin)	NA synthesis inhibitor
60	Topotecan (Hycamtin)	Topoisomerase I inhibitor
61	TM30089	CRTH2-R antagonist
62	Gefitinib (Iressa)	TK inhibitor
63	Etoposide	Topoisomerase II inhibitor
64	Bortezomib (Velcade)	Proteasome inhibitor
65	ABT-737	Bcl-2 inhibitor
66	Sorafenib	TK inhibitor
67	Rapamycin (Sirolimus)	mTOR inhibitor
68	Erlotinib, Hydrochloride	EGF-R inhibitor
69	Paclitaxel (Taxol)	Tubulin stabilizer
70	17-DMAG	HSP 90 inhibitor
71	Sunitinib	TK inhibitor
72	Tandutinib	RTK inhibitor
73	17-AAG	HSP 90 inhibitor

Table S2. Small Molecule Compounds 34-73. Drugs screened within the current study including common name and mode of action.

Disease Models & Mechanisms • Supplementary information

Number	Inhibitor	Activity
74	BI-D1870	p90 RSK inhibitor
75	A-769662	AMPK activator
76	AC220 (Quizartinib)	FLT3 inhibitor
77	AG014699 (PF-01367338)	PARP inhibitor
78	ARRY-162	MEK inhibitor
79	ARQ 197 (Tivantinib)	c-MET inhibitor
80	Atorvastatin Calcium (Lipitor)	HMG-CoA reductase inhibitor
81	AV-951 (Tivozanib)	VEGF-R inhibitor
82	AZD1152	Aurora kinase inhibitor
83	AZD1480	JAK inhibitor
84	AZD4547	FGFR inhibitor
85	AZD8055	mTOR inhibitor
86	Belinostat (PXD101)	HDAC inhibitor
87	BI 6727 (Volasertib)	PLK inhibitor
88	BMS-777607	c-MET inhibitor
89	BSI-201 (Iniparib)	PARP inhibitor
90	CAL-101	PI3K inhibitor
91	Carfilzomib (PR-171)	Proteasome inhibitor
92	CGS 21680	Adenosine receptor agonist
93	CUDC-101	HDAC/EGFR/HER2 inhibitor
94	CYT-387	JAK inhibitor
95	Dutasteride (Avodart)	5α-reductase inhibitor
96	EMD1214063	c-MET inhibitor
97	Eprosartan Mesylate (Teveten)	Angiotensin receptor antagonist
98	GSK1120212	MEK inhibitor
99	GSK461364	PLK inhibitor
100	GSK690693	Akt inhibitor
101	INCB018424 (Ruxolitinib)	JAK inhibitor
102	INK128	mTOR inhibitor
103	Lenalidomide (CC-5013)	Immunomodulator
104	LY294002	PI3K inhibitor
105	MK-4827	PARP inhibitor
106	NVP-LDE225	SMO inhibitor
107	OSI-027	mTOR inhibitor
108	Pazopanib (Votrient)	TK inhibitor
109	PD-0332991	CDK inhibitor
110	PF-04217903	c-MET inhibitor
111	PF-04691502	PI3K/mTOR inhibitor
112	PLX4032 (RG7204)	B-Raf inhibitor
113	PLX4720	B-Raf inhibitor

Table S3. Small Molecule Compounds 74-113. Drugs screened within the current study including common name and mode of action.

Number	Inhibitor	Activity
114	Ponatinib (AP24534)	Bcr-Abl inhibitor
115	Regorafenib (BAY 73-4506)	Multi-kinase inhibitor
116	SR1	AHR antagonist
117	TG100-115	PI3K inhibitor
118	TG101348	JAK inhibitor
119	Tubacin	HDAC inhibitor
120	Tubastatin A	HDAC inhibitor
121	Varespladib (LY315920)	Phospholipase A2 inhibitor
122	VX-765	ICE/Caspase-1 inhibitor
123	VX-950 (Telaprevir)	Protease inhibitor
124	WZ4002	EGF-R inhibitor
125	XL-147	PI3K inhibitor
126	XL-184 (Cabozantinib)	TK inhibitor
127	YM155	Survivin suppressant
128	AS703026 (MSC1936369B)	MEK inhibitor
129	NVP-AUY922 (VER-52296)	HSP 90 inhibitor
130	AZD7762	Checkpoint kinase inhibitor
131	Foretinib	c-MET/VEGFR2 inhibitor
132	Dabrafenib	B-Raf inhibitor
133	GDC-0980	PI3K/mTOR inhibitor
134	NVP-BGJ398	FGFR inhibitor
135	NVP-BKM120	PI3K inhibitor
136	PCI-32765 (Ibrutinib)	Bruton's TK inhibitor
137	Dacomitinib	ErbB-R inhibitor
138	SCH900776	Checkpoint kinase inhibitor
139	VX-11e	ERK2 inhibitor
140	Deforolimus	mTOR inhibitor
141	SGI-1776	Pim-1 inhibitor
142	AZD1208	Pan-Pim inhibitor
APO	Apocynin	Anti-arhtritis/anti-inflammatory
SP	SP600125	MAPL/JNK Inhibitor
OLOM	Olomoucine	CDK2/CDC2/MAPK inhibitor
IV	Butyrolactone I	CDK2/CDC2 inhibitor

Table S4. Small Molecule Compounds 114-142. Drugs screened within the current study including common name and mode of action.

Disease Models & Mechanisms • Supplementary information

Number	Name	Mechanism	IC50 (μM)
70	17-DMAG	HSP 90 inhibitor	0.062
93	CUDC-101	HDAC/EGFR/HER2 inhibitor	0.004/0.0024/0.0157
102	INK-128	mTOR kinase inhibitor	0.001
107	OSI-027	mTORC1 and mTORC2 inhibitor	0.022/0.065
111	PF-04691502	P13K/mTOR inhibitor	0.032

Table S5. Details of Small Molecule Compounds 70, 93 102, 107, 111. Drugs selected for further *in vitro* analysis within the current study including common name, mode of action and IC50 values.

Drug	0.01 µM		0.1 µM		1 µM		10 µM	
	Absorbance	Standard Deviation	Absorbance	Standard Deviation	Absorbance	Standard Deviation	Absorbance	Standard Deviation
70	99.70	5.43	86.84	8.05	83.81	11.69	81.66	15.79
93	103.01	13.01	100.57	2.40	99.90	7.32	97.13	5.61
102	101.19	3.01	85.32	3.27	83.79	8.63	74.22	17.42
107	101.58	5.22	97.44	1.69	82.66	10.00	79.79	17.75
111	96.47	2.41	91.42	8.21	76.47	17.36	81.11	12.28

Table S6. Combined Toxicity Data. Toxicity data from drugs 70, 93, 102, 107 and 111 on human synovial MSCs. Relative (to DMSO control) absorbance data from alamar Blue assay with standard deviation is presented. Boxes highlighted in red represent treatment groups that passed the 85% cutoff selected for acceptable levels of drug induced toxicity. At least 3 independent replicates were performed for each experiment, normal (n=5), OA (n=7).

Disease Models & Mechanisms • Supplementary information

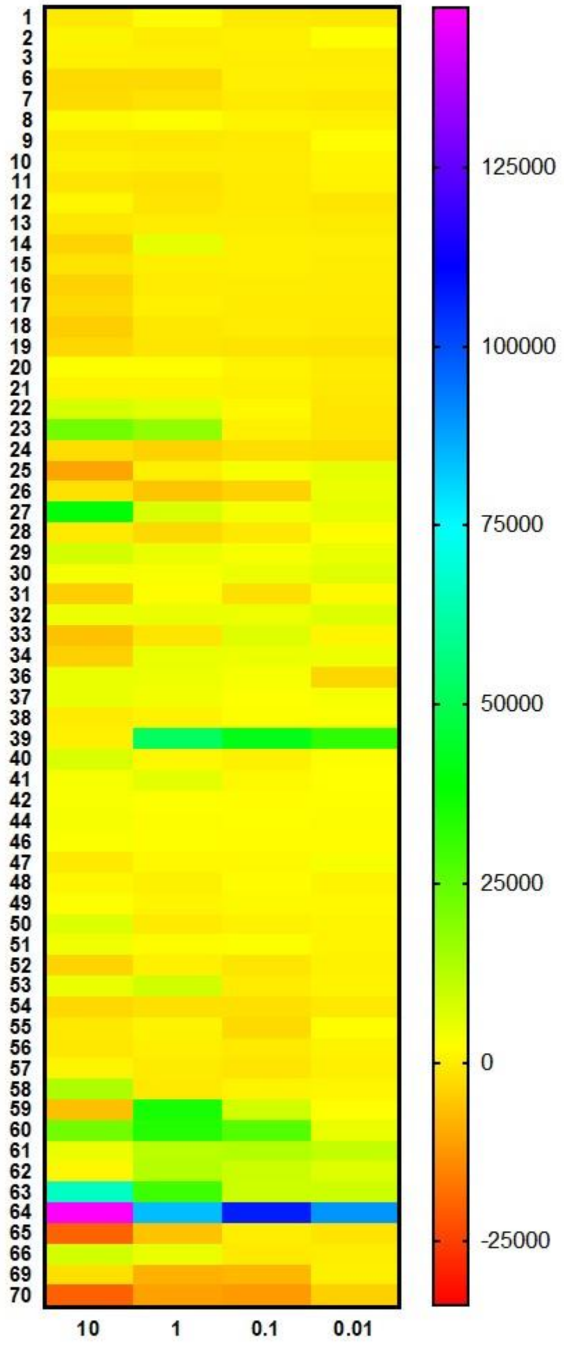

Figure S1. *p21* Expression after Treatment with Small Molecule Compounds 1-70. A heat map representation of *p21* expression levels after treatment with 0.01, 0.1, 1 or 10µM concentrations of drugs 1-70.

Disease Models & Mechanisms • Supplementary information

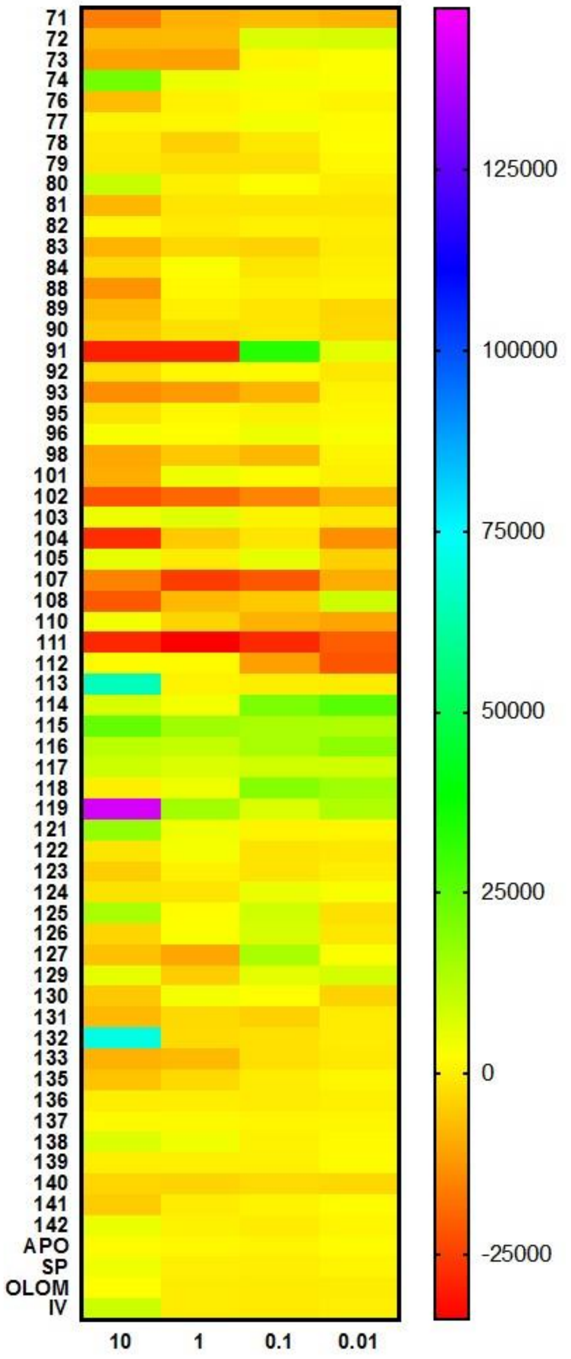

Figure S2. *p21* Expression after Treatment with Small Molecule Compounds 71-142. A heat map representation of *p21* expression levels after treatment with 0.01, 0.1, 1 or 10µM concentrations of drugs 71-142.

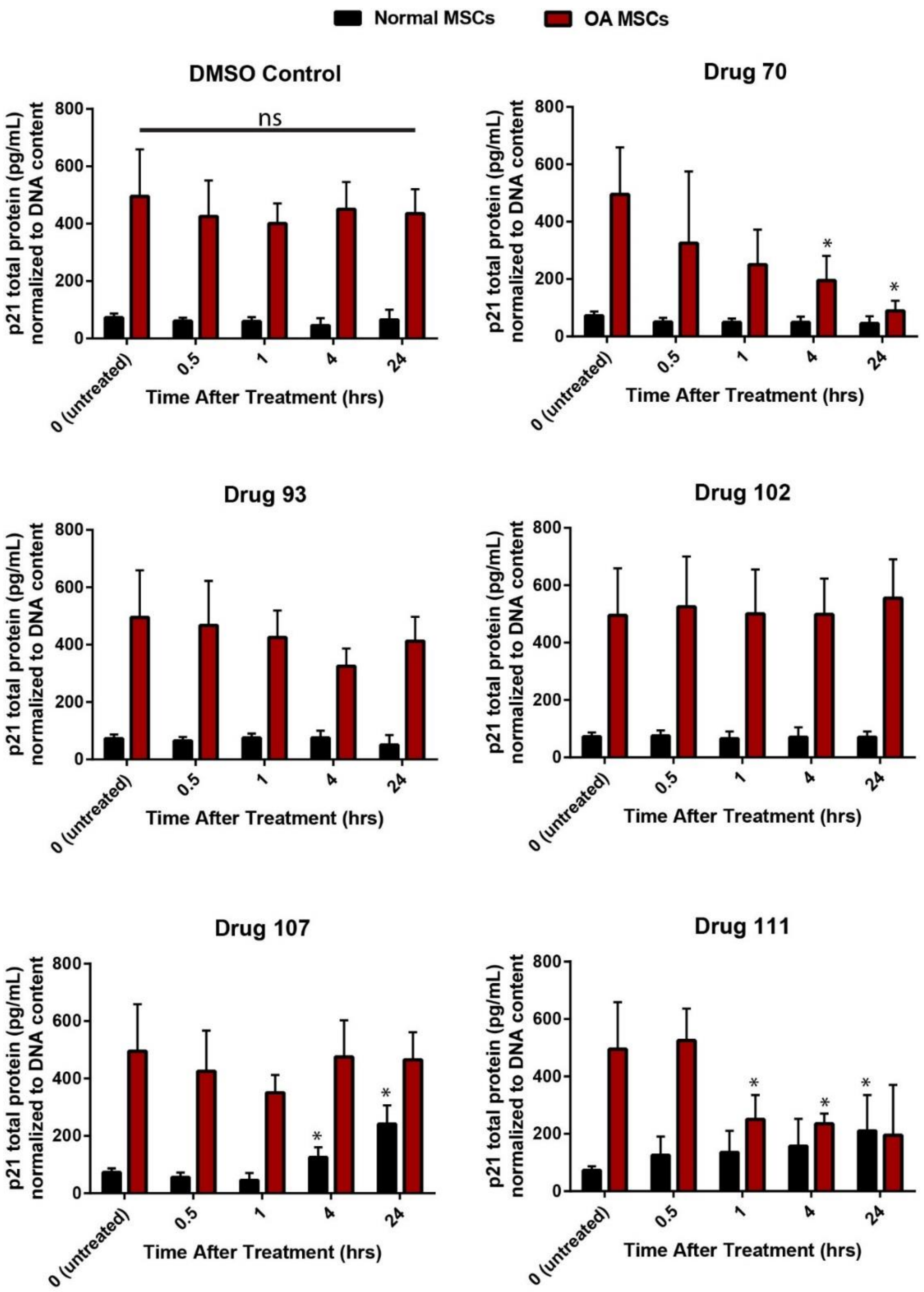

Figure S3. p21 Protein Levels after Drug Treatment. Normal and OA human synovial MSCs were treated with DMSO or drugs 70, 93, 102, 107, 111. p21 protein levels were quantified at 0, 0.5, 1, 4 and 24hrs after treatment. DMSO, drug 93 and 102 had no effect on p21 protein levels, while drug 70 and 111 decreased levels in OA MSCs and Drug 107 and 111 increased p21 levels in normal MSCs. At least 3 independent replicates were performed for each experiment, normal (n=5), OA (n=7). *p<0.05.

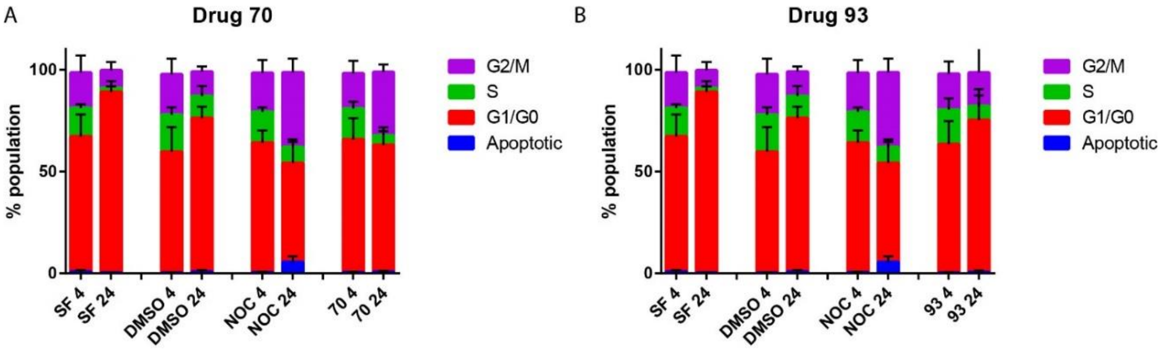

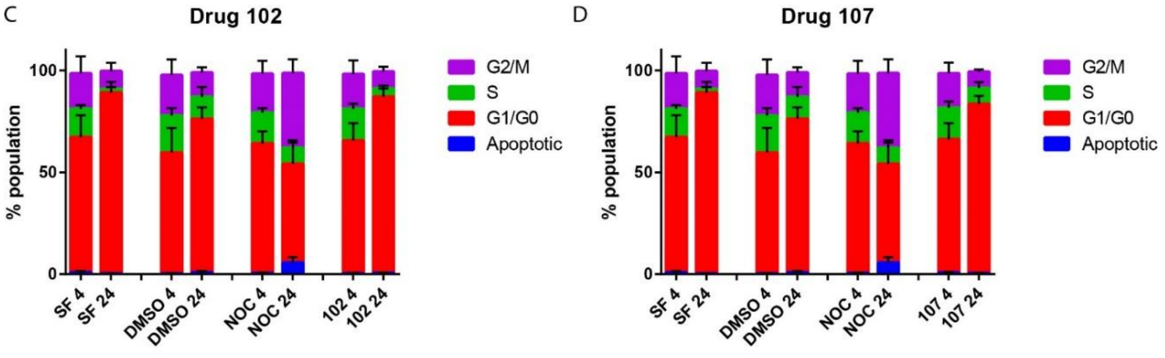

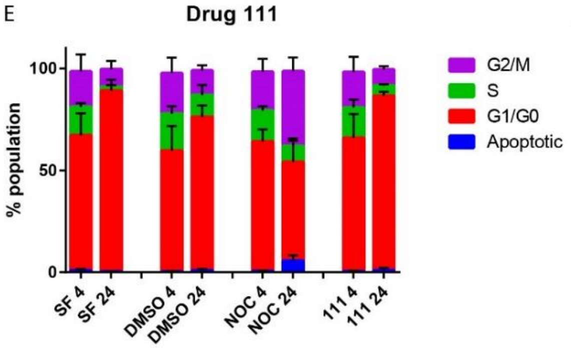

F

% of cells		Apoptotic	G0/G1	S	G2/M
DMSO	4hr	0.3	59.3	18.2	19.9
	24hr	0.9	75.3	10.8	12.0
70	4hr	0.6	65.2	15.1	17.2
	24hr	0.8	62.3	4.6	31.1
93	4hr	0.3	63.1	17.0	17.5
	24hr	0.7	74.5	6.9	16.4
102	4hr	0.4	65.2	15.6	17.0
	24hr	0.7	86.4	4.0	8.3
107	4hr	0.7	65.5	15.4	16.9
	24hr	0.5	83.0	7.8	7.9
111	4hr	0.5	65.5	14.8	17.4
	24hr	0.9	85.7	5.0	7.8

Figure S4. Expanded Cell Cycle Data. Cell cycle data from drugs 70 (A), 93 (B), 102 (C), 107 (D) and 111 (E) on human synovial MSCs. The percentage of cells in G2/M, S, G1/G0 or undergoing apoptosis was assayed for each drug treatment at 4 and 24hrs post treatment. DMSO was employed as a negative control, while cells cultured under serum free (SF) were used to control for G0/G1 accumulation and nocodazole (NOC) for G2/M accumulation. Overall, drug 70 increased the percentage of cells in G2/M by 24hrs and drugs 102 and 111 increased the percentage of cells in G0/G1 by 24hrs post treatment (E). Boxes highlighted in red represent significance ($p<0.05$). At least 3 independent replicates were performed for each experiment.

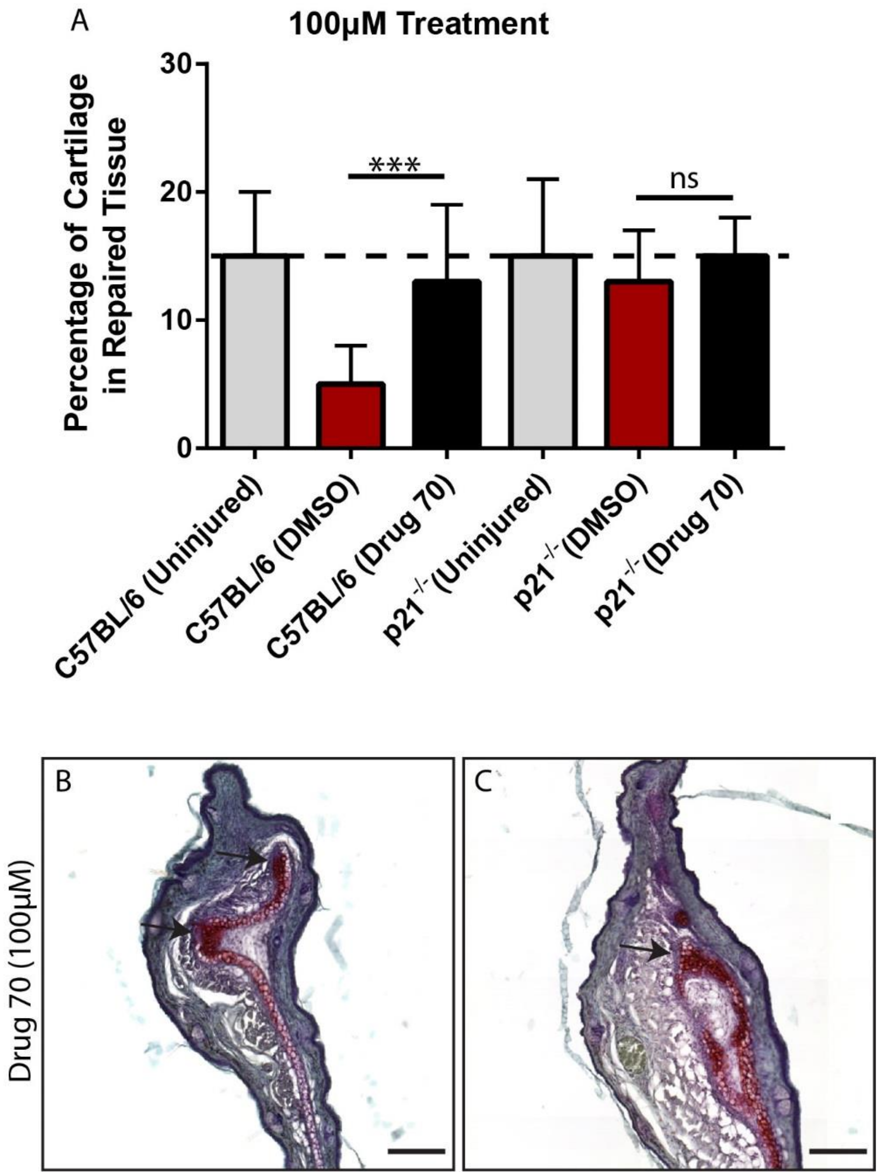

Figure S5. At a higher concentration of drug 70 (100µM), increased cartilage formation is still observed (A)(arrows, B,C), however, this concentration negatively affected surrounding tissue morphology. Scale bars equal 200µm, *** = p<0.01.

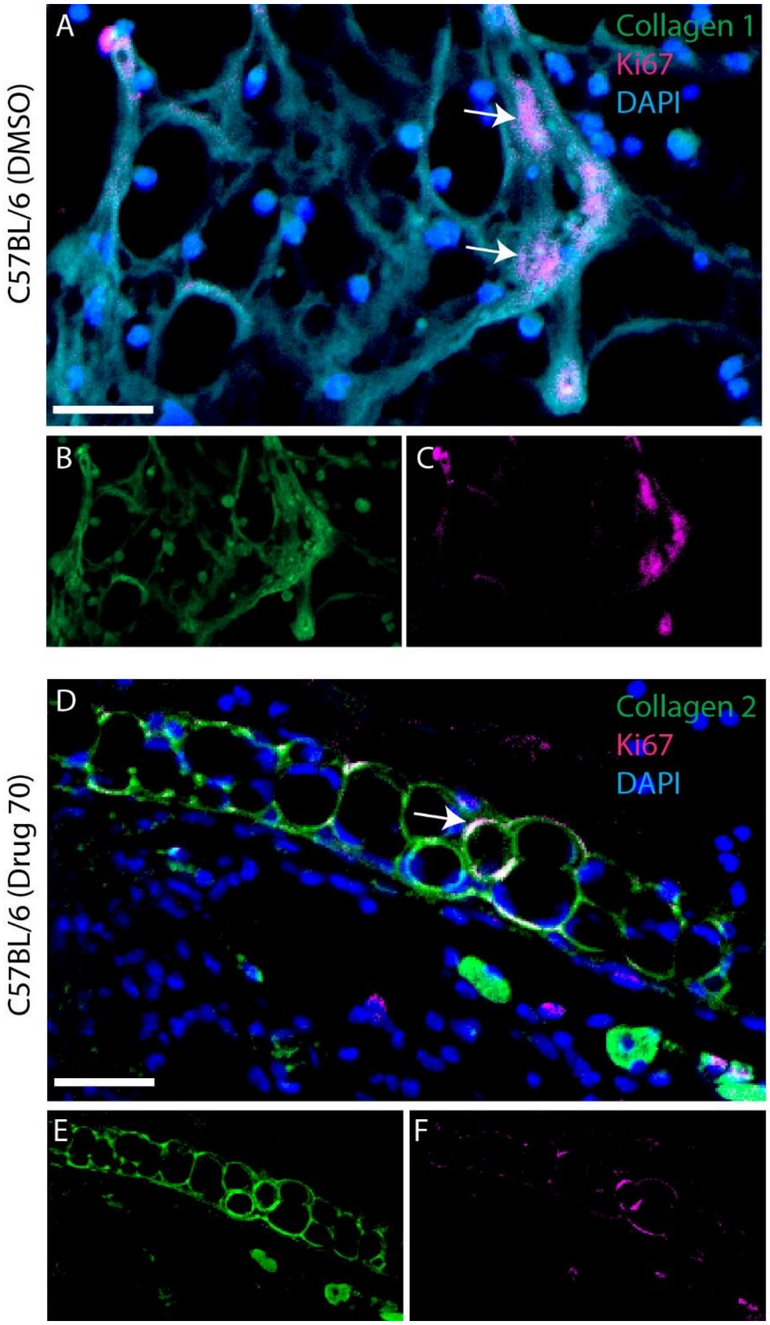

Figure S6. Ki67 Co-localization with Collagen Staining in DMSO and Drug 70 Treated Injuries. In C57BL/6 mice with DMSO treated injuries (A-C) the cells within the collagen scaffold of the ear begin to proliferate (C) but lose expression of collagen type 2 and instead express collagen type 1 (B). When treated with drug 70 (D-F), cells begin to proliferate (F), but maintain collagen type 2 expression (E). Scale bars equal 50µm.
